# High‐throughput custom capture sequencing identifies novel mutations in coloboma‐associated genes: Mutation in DNA‐binding domain of retinoic acid receptor beta affects nuclear localization causing ocular coloboma

**DOI:** 10.1002/humu.23954

**Published:** 2019-12-09

**Authors:** Vijay K. Kalaskar, Ramakrishna P. Alur, LeeAnn K. Li, James W. Thomas, Yuri V. Sergeev, Delphine Blain, Robert B. Hufnagel, Tiziana Cogliati, Brian P. Brooks

**Affiliations:** ^1^ Pediatric, Developmental & Genetic Ophthalmology Section, Ophthalmic Genetics and Visual Function Branch (OGVFB), National Eye Institute (NEI) National Institutes of Health (NIH) Bethesda Maryland; ^2^ National Institutes of Health Intramural Sequencing Center National Human Genome Research Institute, NIH Bethesda Maryland; ^3^ Protein Biochemistry and Molecular Modeling Group, OGVFB, NEI NIH Bethesda Maryland; ^4^ Ophthalmic Clinical Genetics Section, OGVFB, NEI NIH Bethesda Maryland; ^5^ Medical Genetics and Ophthalmic Genomics Unit, OGVFB, NEI NIH Bethesda Maryland

**Keywords:** coloboma, custom capture, high‐throughput sequencing, retinoic acid receptor beta

## Abstract

Uveal coloboma is a potentially blinding congenital ocular malformation caused by the failure of optic fissure closure during the fifth week of human gestation. We performed custom capture high‐throughput screening of 38 known coloboma‐associated genes in 66 families. Suspected causative novel variants were identified in *TFAP2A* and *CHD7*, as well as two previously reported variants of uncertain significance in *RARB* and *BMP7*. The variant in *RARB*, unlike previously reported disease mutations in the ligand‐binding domain, was a missense change in the highly conserved DNA‐binding domain predicted to affect the protein's DNA‐binding ability. In vitro studies revealed lower steady‐state protein levels, reduced transcriptional activity, and incomplete nuclear localization of the mutant RARB protein compared with wild‐type. Zebrafish studies showed that human *RARB* messenger RNA partially reduced the ocular phenotype caused by morpholino knockdown of *rarga* gene, a zebrafish homolog of human *RARB*. Our study indicates that sequence alterations in known coloboma genes account for a small percentage of coloboma cases and that mutations in the *RARB* DNA‐binding domain could result in human disease.

## 1 INTRODUCTION

Uveal coloboma is an ocular developmental malformation caused by the failure of the ventral optic fissure to close (ALSomiry, Gregory‐Evans, & Gregory‐Evans, 2019; Gregory‐Evans, Williams, Halford, & Gregory‐Evans, 2004; Onwochei, Simon, Bateman, Couture, & Mir, 2000; L. Wang et al., 2012). Different parts of the eye may be affected including the iris, ciliary body, neurosensory retina, retinal pigment epithelium (RPE) and choroid, with or without the involvement of the optic nerve. Coloboma accounts for up to 10% of pediatric blindness worldwide, with a prevalence of five to seven cases per 100,000 births (Bermejo & Martinez‐Frias, 1998; Chang, Blain, Bertuzzi, & Brooks, 2006; Lu, 1989; Onwochei et al., 2000; Stoll, Alembik, Dott, & Roth, 1992). Ocular complications include decreased best‐corrected visual acuity of varying severity, reduced visual field, photoaversion, and increased risk for retinal detachment, choroidal neovascularization, and cataracts (Chang et al., 2006; Onwochei et al., 2000). Syndromic cases of inherited coloboma can present with other defects such as spinal abnormalities, kidney and heart malformations, and central nervous system defects (Chang et al., 2006; Gregory‐Evans, Vieira et al., 2004; Huynh et al., 2013; L. Wang et al., 2012).

Uveal coloboma is believed to have a significant genetic component; however, the genes and mutations behind the majority of coloboma cases currently remain unknown (Gregory‐Evans, Vieira et al., 2004; Morrison et al., 2002; L. Wang et al., 2012). The presence of multiple patterns of Mendelian inheritance, incomplete penetrance, variable expressivity, genetic heterogeneity, and environmental factors make the genetic investigation of this disease particularly challenging (Chang et al., 2006; Skalicky et al., 2013; Williamson & FitzPatrick, 2014). Although several genes identified in animal studies are critical for optic fissure closure during eye development, very few are known to cause coloboma in humans (Brown et al., 2009; Patel & Sowden, 2019). On the other hand, reports of human mutations are often not complemented by experimental mechanistic evidence either in animal models or in cell culture (Bourchany et al., 2015; Chassaing et al., 2016; Graham et al., 2003; Kahrizi et al., 2011; Martinez‐Garay et al., 2007; Ng et al., 2012; Patel & Sowden, 2019; Wenger et al., 2014). In many cases, studies on coloboma genetics have focused on a select number of genes, and many have included syndromic conditions with severe ocular phenotypes (e.g., severe microphthalmia and anophthalmia; Gonzalez‐Rodriguez et al., 2010; Guo, Dai, Huang, Liao, & Bai, 2013; Mihelec et al., 2009; Morrison et al., 2002; Schimmenti et al., 2003; Williamson & FitzPatrick, 2014; X. Zhang et al., 2009; J. Zhou et al., 2008).

Phenotypic variability in ocular conditions likely depends on multiple factors, including genetic variation in coding and noncoding elements in and around genes important for orchestrating ocular development (Azuma et al., 2003; Chang et al., 2006; George et al., 2016; Reis & Semina, 2015; Sanyanusin et al., 1995). Mutations leading to partial or complete loss of gene function have been shown to cause severe ocular and nonocular systemic manifestations in addition to coloboma (Asai‐Coakwell et al., 2009; Beleggia et al., 2015; C. Liu et al., 2016; Reis & Semina, 2015; Ye et al., 2010). Genes that encode transcriptional regulators (e.g., *PAX2*, *PAX6*, *MITF*, *TFAP2A*, and *CHD7*), members of secreted signaling pathways, such as transforming growth factor‐beta/bone morphogenetic protein (TGFB/BMP) signaling (*BMP7*, *GDF3*, and *GDF6*), Hippo signaling (*YAP1*), retinoic acid (RA) signaling (*ALDH1A3*, *STRA6*, and *RARB*) and those involved in membrane transport (*ABCB6*) have been implicated in causing coloboma when mutated in humans (Reis & Semina, 2015). Transcription factors such as RAX, SOX2, PAX6, PAX2, MITF, and OTX2 are expressed very early during development and are involved in eye field specification and emergence of the optic vesicle, cell lineage specification, and development of the neural retina and RPE (Avilion et al., 2003; Chow & Lang, 2001; Levine & Brivanlou, 2007; Zuber, Gestri, Viczian, Barsacchi, & Harris, 2003). Secreted signaling molecules regulate the spatio‐temporal expression of multiple transcription factors and play a critical role in the morphogenesis of the optic cup and the establishment of RPE and retinal domains (Behesti, Holt, & Sowden, 2006; Kobayashi, Yasuda, & Araki, 2010; Zhao, Saitsu, Sun, Shiota, & Ishibashi, 2010). Members of TGFB/BMP, WNT, and Sonic hedgehog (SHH) signaling are involved in dorsal–ventral patterning of the retina and RPE by regulating the expression of T‐box (*Tbx5*, *Tbx3*, and*Tbx2*), *Vax*1/2, *Msx1*, *Pax6*, and *Pax2* genes (Behesti et al., 2006; Kobayashi et al., 2010; Zhao et al., 2010). WNT, TGFB/BMP, and Hippo signaling pathways have been shown to regulate RPE specification, development, and choroid fissure closure (Fossdal et al., 2004; Fuhrmann, Levine, & Reh, 2000; Miesfeld et al., 2015; Muller, Rohrer, & Vogel‐Hopker, 2007; Steinfeld et al., 2013; Williamson et al., 2014). Fibroblast growth factor signaling has been shown to induce and maintain the neural identity of the retina (Hyer, Mima, & Mikawa, 1998; Pittack, Grunwald, & Reh, 1997). Genes associated with SHH signaling have been shown to be expressed in the neural retina and other ocular tissues during development (Amato, Boy, & Perron, 2004; Wallace, 2008). Mutations in humans and studies with specific misexpression and loss‐of‐function of SHH genes have been associated with coloboma and other severe ocular defects (Schimmenti et al., 2003; X. M. Zhang & Yang, 2001). TGFB/BMP signaling genes have been shown to be expressed in all ocular tissues including the periocular mesenchyme and reports in humans (e.g., *BMP4*) and animal studies suggest a role in ocular, brain, craniofacial, and skeletal abnormalities (Bakrania et al., 2008; Furuta & Hogan, 1998). Mutations in the WNT receptor, FZD5 have been associated with optic fissure closure defects and were shown to disrupt the apical junctions of the retinal neural epithelium leading to coloboma (C. Liu et al., 2016). The complexity of the interactions between the signaling pathways and the transcription factors involved in patterning and development of the eye, however, remains only in part understood.

Among the signaling pathways, RA signaling has been well characterized for its role in ocular development, and mutations in several genes associated with RA signaling have been reported to cause coloboma in human and animal models (Jakubiuk‐Tomaszuk et al., 2019; Lupo et al., 2011; Matt et al., 2005; Matt, Ghyselinck, Pellerin, & Dupe, 2008; Srour et al., 2013, 2016). RA temporal and spatial expression in the developing eye is tightly regulated through the balance of synthesis, controlled by retinol and retinaldehyde dehydrogenases, and degradation by cytochrome P450 (Rhinn & Dolle, 2012). In the cytoplasm, RA binds to cellular RA‐binding proteins (CRABP1/2) and upon entering the nucleus, RA binds to heterodimer receptor complexes comprising nuclear RA receptors (RARA/B/G) and retinoid X receptors (RXRA/B/G) to regulate the expression of target genes (Cvekl & Wang, 2009; Kastner et al., 1997; Kedishvili, 2013).

During embryonic development, RA signaling has been shown to have a predominant role in eye morphogenesis and optic fissure closure (Gestri, Bazin‐Lopez, Scholes, & Wilson, 2018; Matt et al., 2008). Several genes associated with RA signaling, such as retinoid receptors, *RAR*s (A, B, and G) and *RXR*s (A, B, and G), *STRA6*, and RA‐synthesizing enzymes, *RALDH1* and *RALDH3*, are expressed in the developing eye and the surrounding periocular mesenchyme (Bouillet et al., 1997; Ghyselinck et al., 1997; Matt et al., 2005; Mori, Ghyselinck, Chambon, & Mark, 2001). Retinoid nuclear receptors are the key transducers of RA signaling and have been shown to heterodimerize to regulate their target genes (Kastner et al., 1997). The role of retinoid receptors has been investigated in single, double, and multigene knockout animal models (Ghyselinck et al., 1997; Grondona et al., 1996; Kastner et al., 1997; Lohnes et al., 1994; Mendelsohn et al., 1994). *Rarb* knockout mice look overall morphologically normal; however, they show ocular defects including retrolenticular membrane with persistent hyaloid vasculature, congenital retinal folds, cataracts, reduced eye size and weight, and decreased retinal ganglion cell number (Ghyselinck et al., 1997; G. Zhou, Strom, Giguere, & Williams, 2001). In contrast, compound mutants of *Rarb* gene with other receptor genes, *Rara* or *Rarg*, exhibit developmental defects in several organ systems leading to embryonic lethality during late gestation. *Rarb* and *Rara* or *Rarg* compound mutant mouse embryos display severe ocular defects including retinal dysplasia and degeneration, shortening of the ventral retina, absence of the anterior chamber, and ventral rotation of the lens (Ghyselinck et al., 1997; Grondona et al., 1996; Lohnes et al., 1994; Mendelsohn et al., 1994). These studies underscore the importance of *RARB* and its heterodimerization partners in correctly transducing the RA signaling during ocular morphogenesis.

In recent reports, patients carrying *RARB* mutations are described with a severe syndromic phenotype affecting several organ systems and including coloboma, microphthalmia, anophthalmia, cardiac defects, progressive motor impairment, pulmonary hypoplasia, and diaphragmatic hernia. The severity of these phenotypes is reported to be a consequence of either a total loss‐of‐function or two‐ to threefold gain‐of‐function of the *RARB* gene (Srour et al., 2013, 2016). The molecular mechanisms underlying the multitude of phenotypes observed in individuals with *RARB* mutations are not clear.

In the present study, we report on the screening of a large cohort of syndromic and nonsyndromic uveal coloboma patients using custom capture sequencing and on the identification of novel mutations in genes previously associated with coloboma. We further explore the mechanisms underlying the mutation in *RARB* DNA‐binding domain that resulted in structural and functional changes within the RARB protein ultimately affecting RA signaling.

## 2 MATERIALS AND METHODS

### 2.1 Subjects

Two hundred twenty‐eight study subjects (99 affected) from 66 families, including probands with primarily syndromic or nonsyndromic coloboma and their first‐degree relatives, were examined by a single ophthalmologist (BPB) at the National Eye Institute (NEI) Ophthalmic Genetics Clinic between April 2004 and June 2013 under Institutional Review Board (IRB)‐approved protocols (National Institutes of Health database, clinicaltrials.gov, identifiers: NCT00076271, NCT00368004, NCT01778543, and NCT000708955). Informed consent was obtained from patients and/or their parents, under the approval of the NEI IRB or the NIH Combined Neuroscience Institutional Review Board, depending on the time frame. Trios for each of the families were assessed at a minimum, with further members, affected and unaffected, included for 17 of the families. For probands, a complete systemic work‐up tailored to phenotypic associations to coloboma was obtained (either from medical records or at the NIH Clinical Center; Huynh et al., 2013).

### 2.2 Custom capture sequencing and mutation detection

Genomic DNA was extracted from whole blood or saliva. Two custom capture designs (CC‐1 and CC‐2) were used for targeted sequencing covering a total of 193 genes (Table S1). The first (CC‐1) was created using the Illumina Design Studio to design TruSeq Custom Enrichment Oligos targeting the RefSeq exon annotation (GRCh37, protein‐coding and untranslated regions [UTRs] including the promoter region) covering 167 genes. The second design (CC‐2) was also based on RefSeq exon annotation (protein‐coding and UTRs including the promoter region) and created using the NimbleDesign software for SeqCap® EZ Choice probes (Roche) targeting 193 genes. Indexed libraries were constructed, pooled, captured, and then sequenced together on either the Illumina HiSeq 2000 (v3 reagents, 101‐base paired‐end reads) or the HiSeq 2500 (v4 reagents, 126‐base paired‐end reads). The first stage custom capture (CC‐1) sequencing included 42 families and analysis was performed on 34 families which had at least trios with 110 study subjects. The second stage (CC‐2) included another 42 families with eight families from the first stage and analysis was performed on 40 families with 143 study subjects.

Sequence reads were aligned to the human reference genome hg19 (GRCh37) using Novoalign V2.08.02. Polymerase chain reaction (PCR) duplicates were removed with Samtools (https://www.ncbi.nlm.nih.gov/pubmed/19505943). A minimum coverage threshold of 90% was required and samples with lower coverage were resequenced to improve the coverage. Variant calling was performed, and quality scores computed by MPG (“Most Probable Genotype” caller) algorithm (https://www.ncbi.nlm.nih.gov/pubmed/20810667). Only variants with an MPG score >10 and an MPG score to the depth of coverage ratio >0.5 were examined. Variants were annotated using ANNOVAR and the UCSC Known Gene model.

Variant results were analyzed and filtered in VarSifter‐based multitiered screening (Teer, Green, Mullikin, & Biesecker, 2012). The first‐pass filter included the following criteria for variant quality screening and filtering: ClinSeq reference allele is the major allele and present at >50% frequency in the population, ClinSeq variant (minor) allele frequency ≤0.02 is considered and genotypes called when >25 samples in the cohort covered for the location (Biesecker et al., 2009). The variants obtained were further filtered out by comparing with other population databases, such as 1000 Genomes, Exome Variant Server (http://evs.gs.washington.edu/EVS/) and gnomAD (Genome Aggregation Database; gnomad.broadinstitute.org). Pathogenicity and deleteriousness of the variant was predicted using different algorithms: Combined Annotation Dependent Depletion (http://cadd.gs.washington.edu/), Polyphen‐2 (Polymorphism Phenotyping v2; http://genetics.bwh.harvard.edu/pph2/index.shtml), Sorting Intolerant From Tolerant (http://sift.bii.a-star.edu.sg/) and MutationTaster (http://www.mutationtaster.org/; Adzhubei et al., 2010; Kircher et al., 2014; Kumar, Henikoff, & Ng, 2009; Schwarz, Rodelsperger, Schuelke, & Seelow, 2010). The following reference sequences were used for the variants reported in this study: *RARB*: NM_001290216.2, NP_001277145.1; *BMP7*: NM_001719.2, NP_001710.1; *TFAP2A*: NM_003220.2; *CHD7*: NM_017780.3, NP_060250.2.

### 2.3 Cell cultures and plasmid DNA constructs

Human embryonic kidney 293 (HEK 293) cells were cultured in Dulbecco's modified Eagle's medium containing 1% each of l‐glutamine, sodium pyruvate, and antibiotics (penicillin/streptomycin: 10,000 units/ml/10,000 µg/ml) and 10% fetal bovine serum (FBS) as previously described (George et al., 2016). For experiments involving RA treatment, cells were cultured in medium containing 10% FBS or charcoal‐stripped serum for about 24 hr before RA treatment. pcDNA3.1+‐DYK‐tagged *RARB* plasmid DNA (DYK‐tag sequence at C‐terminus; Clone ID: OHu15136) was obtained from GenScript, site‐directed mutagenesis was performed to generate the *RARB*‐mutant plasmid DNA and verified by sequencing. *RARB* and *RARB‐*mutant complementary DNA (cDNA) were cloned in‐frame with a green fluorescent protein (GFP) into a pAcGFP1‐C3 plasmid vector (GFP at C‐terminus). *RXR*A, *RXR*G, and *RAR*G cDNA were cloned in‐frame with RFP into a pAcRFP1‐C3 plasmid vector (RFP at C‐terminus) and used in cotransfection studies. Cells were transfected at 60–70% confluency with plasmid DNA (9 nM) using Extreme HP transfection reagent (Cat # 6366236001; Sigma‐Aldrich) followed by RA treatment (10 µM) after 12 hr. Cells were fixed or harvested 12 hr after RA treatment for further analysis.

### 2.4 Immunofluorescence and imaging

Immunofluorescence staining was performed following a standard protocol as previously described (George et al., 2016). HEK 293 cells on slides (Nunc Lab‐Tek II Chamber slide system, Cat # 154526; MG Scientific) were washed with 1X phosphate‐buffered saline (PBS) and fixed in 4% paraformaldehyde for 10 min followed by three 5‐min washes with 1X PBS and stored at −30°C until used. Slides were blocked in 1X PBS/10% donkey serum/1% bovine serum albumin (BSA)/0.1% Tween20 for 1 hr followed by overnight incubation at 4°C with primary antibody diluted in blocking solution. Slides were washed five to six times after primary antibody incubation with wash buffer containing 1X PBS/1% BSA/0.1% Tween20. Slides were then incubated in secondary antibody with Hoechst (1:2500, Cat # H3570; Invitrogen) for 1 hr at room temperature followed by washings and mounting in Fluormount‐G (Cat # 00100‐01; Southern Biotech). The following antibodies and dilutions were used: rabbit anti‐DYK Flag (1:100, Cat # 14793; Cell Signaling), rabbit polyclonal anti‐RARB (1 µg/ml, Cat # ab53161; Abcam), donkey anti‐rabbit Alexa 555 (1:1,000). Images were taken on Zeiss LSM 700 and LSM 800 confocal microscopes. Z‐stack images were taken with ×63/1.4 Oil Plan‐Apochromat objective with a scan area of *X*: 0.5, *Y*: 0.5 and the following parameters: Z‐stack range 6–14 µm; image scaling (*X*) 0.198 × (*Y*)0.198 × (*Z*)0.30 µm; bit depth −16 bit; image size 1,024 × 1,024 pixels; bidirectional scanning with averaging at 2. At least three to five different single plane fields for each specific staining were used for statistical analysis and single‐plane images were used to generate figure panels. Statistical significance for the cell counts was determined by one‐way analysis of variance test followed by Tukey's multiple comparison tests.

### 2.5 Western blot analysis

Cultured HEK 293 cells were harvested for protein in radioimmunoprecipitation assay (RIPA) buffer (Cat # 89901; Invitrogen) containing protease inhibitors cocktail (REF # 04693159001; Roche). Protein concentration was estimated with the bicinchoninic acid (BCA) method following manufacturer protocol (Cat # 23227; Thermo Fisher Scientific). Nuclear and cytoplasmic fractions were separated as previously described (George et al., 2016) using manufacturer protocol (Cat # 40010; Active Motif). Proteins were separated on AnykD Criterion TGX Precast Midi Protein Gels (Cat # 5671123; Bio‐Rad) followed by transfer onto polyvinylidene difluoride (PVDF) membrane using Trans‐Blot Turbo Transfer System (Cat # 1704150, 1704275; Bio‐Rad). Blots were incubated with antibodies using iBind Flex Western Processor with iBind Flex Cards and Fluorescent Detection Solution kit (Cat # SLF2000, SLF2019, SLF2010; Invitrogen) per manufacturer protocol. The following antibodies and dilutions were used: rabbit monoclonal anti‐DYK Flag (1:1,000, Cat # 14793; Cell Signaling Technology), mouse monoclonal anti‐FLAG M2 antibody (1:1,000, Cat # F3165; Sigma‐Aldrich), rabbit polyclonal anti‐RARB (1 µg/ml, Cat # ab53161; Abcam), rabbit polyclonal anti‐GFP (1:2,500, Cat # ab290; Abcam), rabbit polyclonal anti‐RFP antibody (1:4,000, Cat # R10367; Thermo Fisher Scientific), IRDye 680LT goat anti‐rabbit immunoglobulin G (IgG) (H+L) (1 µl/4 ml, Cat # P/N 926–68021; Li‐COR Bio), IRDye 680RD goat anti‐mouse IgG (H+L) (1 µl/4 ml, Cat # P/N 926–68070; Li‐COR Bio), IRDye 800CW goat anti‐mouse IgG (H+L) (1.3 µl/4 ml, Cat # P/N 926–32210; Li‐COR Bio), IRDye 800CW goat anti‐rabbit IgG (H+L) (1.3 µl/4 ml, Cat # P/N 925–32211; Li‐COR Bio), mouse monoclonal anti‐β‐actin (ACTB; 1:4,000, Cat # A2228; Sigma‐Aldrich). Stripping was done using NewBlot PVDF Stripping Buffer (Cat # 928‐40032; Li‐COR Bio). Images were captured on a Bio‐Rad ChemiDoc imaging system (Cat # 17001402). Western blots were performed on at least six biological replicates of whole‐cell protein extracts and at least three biological replicates of nuclear–cytoplasmic protein fractions. At least two technical repeats were performed for each experiment. Densitometric analysis was performed using the Bio‐Rad's Image Lab 6.0.1 software.

### 2.6 Protein degradation assay

Protein degradation assay was performed as previously described (Alur et al., 2010). HEK 293 cells in culture were treated with 100 µg/ml of cycloheximide (dissolved in 100% ethanol) after 24 hr of transfection. Cells were harvested before and at 3, 6, and 12 hr after treatment. For control, transfected cells were treated with an equal volume of 100% ethanol (vehicle). At least three replicate protein samples were collected for each of the time points and western blot analysis repeated two times.

### 2.7 Luciferase assays

Renilla luciferase reporter plasmid DNA (pRL‐TK) and *CYP26A1* promoter region cloned firefly luciferase reporter plasmid DNA (pGL4.10) were cotransfected with pcDNA3.1+‐DYK‐tagged *RARB* or *RARB*‐mutant plasmids into HEK 293 cells in culture. Genomatix genome analyzer software was used to identify the binding sites for RARB protein in the *CYP26A1* promoter region. The cultured cells were lysed in passive lysis buffer and samples prepared as per manufacturer protocol using the Dual‐Luciferase Reporter assay system (Cat # E1960; Promega). Luminescence was measured using a Modulus microplate multimode reader (Model # 9300‐010; Turner Biosystems). The experiments were performed at least six times with three replicates for each sample luminescence reading. Statistical significance was determined by paired *t* test. Error bars represent standard error of the mean (*SEM*).

### 2.8 Zebrafish experiments

Morpholinos targeting the translation start site (CCAGAGCCTCCATACAGTCGAACAT) and splice site (CTGGCAGAGGTCTAAACCCTCACCT) of the *rarga* gene were obtained from GeneTools LLC. Morpholinos were injected into 1–2 cell‐stage zebrafish embryos at different concentrations and rescue experiments conducted using human *RARB* and *RARB*‐mutant messenger RNA (mRNA). The embryos were imaged under a dissecting microscope at 24, 48, and 72 hr postfertilization (hpf). The effect of morpholino knockdown was determined by quantitative real‐time polymerase chain reaction (qRT‐PCR). Morpholino injections were performed at least six different times for each morpholino and the mRNA rescue experiments repeated at least five times.

### 2.9 Quantitative real‐time polymerase chain reaction

RNA was prepared from zebrafish embryos or HEK 293 cells using NucleoSpin RNA isolation kit (Cat # 740955.50; Macherey‐Nagel) and cDNA synthesized using SuperScript First‐strand synthesis system for RT‐PCR (Cat # 11904018; Thermo Fisher Scientific). TaqMan qPCR assay was performed using Universal Probe Library probes (REF # 04683633001, 04869877001; Roche). At least three different biological samples, each with three technical replicates were performed. Transfected *RARB* expression was determined by using two primer sets (one for the N‐terminus and one for the C‐terminus) and normalized to housekeeping genes *GAPDH* or *PPIA* and to the neomycin‐resistance gene expressed from the plasmid vector. For zebrafish qPCR experiments, *actb1* or *rpl13a* were used as housekeeping control genes. Statistical significance was determined by paired *t* test. Error bars represent *SEM*.

## 3 RESULTS

### 3.1 Custom capture sequencing identifies novel mutations

**Figure 1:**
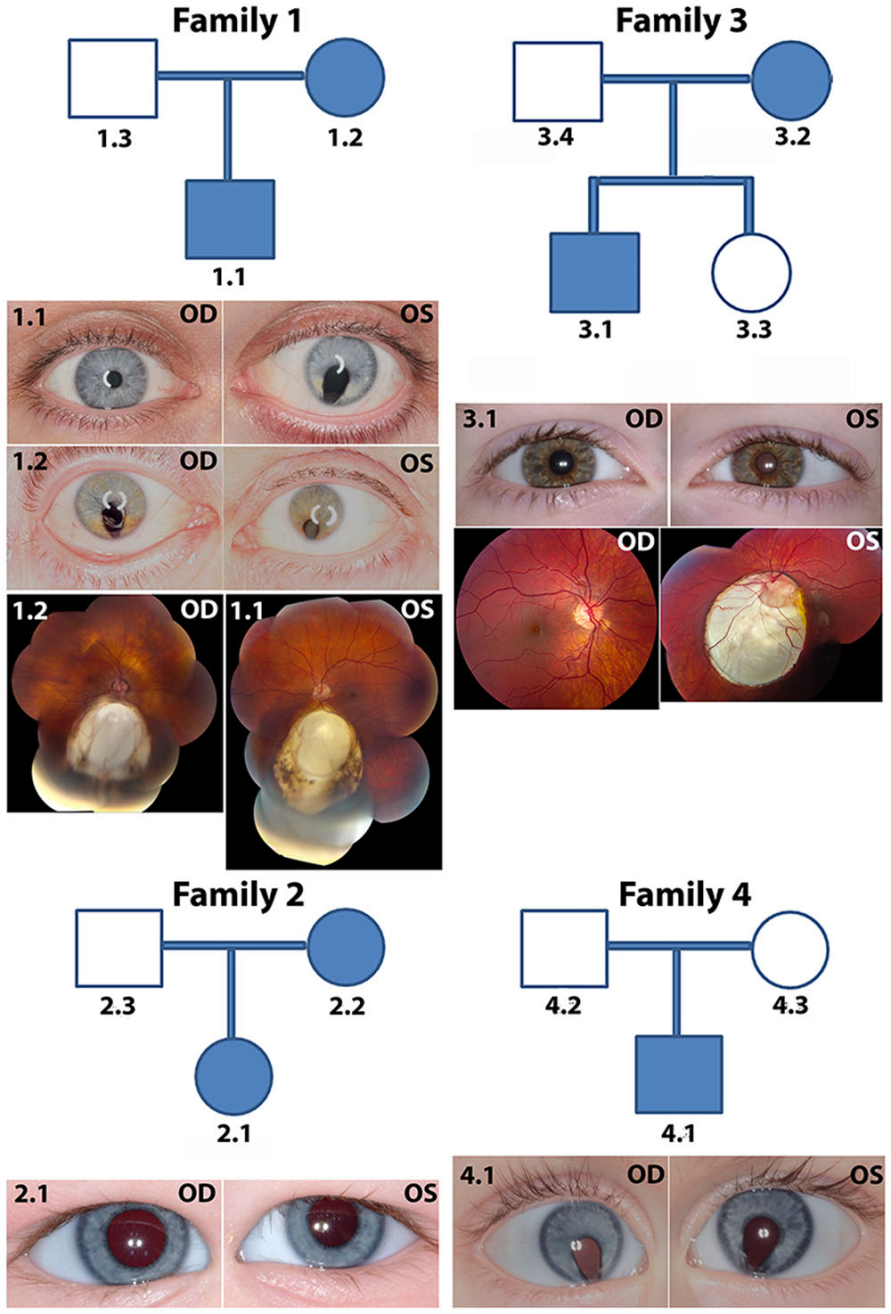
Clinical findings in four coloboma families. Family 1: The proband (1.1) had bilateral coloboma in the retina and iris coloboma in the left eye; the mother (1.2) presented with left eye microcornea and posterior coloboma and right eye with chorioretinal coloboma inferior to the optic disc. Family 2: The proband (2.1) had left eye microcornea and coloboma in the optic nerve and inferior retina. Family 3: The proband (3.1) had bilateral coloboma. Family 4: The proband (4.1) had bilateral chorioretinal coloboma; the right eye was microphthalmic. OD, right eye; OS, left eye

Custom capture sequencing was performed in two stages, involving a total of 228 individuals (99 affected) from 66 families. The sequencing panels combined covered exonic regions corresponding to 38 known coloboma‐associated genes (Table S1). These genes have been reported to cause human coloboma, microphthalmia, and/or anophthalmia (Brown et al., 2009; Chow & Lang, 2001; Cvekl & Tamm, 2004; Fuhrmann, 2010; Lang, 2004; Martinez‐Morales, Rodrigo, & Bovolenta, 2004; Zuber et al., 2003). The first‐pass filter eliminated most of the common variants and passed rare variants including, missense, nonsense, frameshift, and splice site in each of the families. Variants were further filtered out by cross‐verification with individual BAM files and comparing with population databases and deleteriousness prediction using freely available web‐based algorithms. The final candidate variants obtained were verified by Sanger sequencing to check if they correctly segregated with the affected individuals in each of the families. Of the 38 known coloboma‐associated genes, the analysis identified novel variants in two genes, *CHD7*, *TFAP2A*, and previously reported variants in *RARB* and *BMP7*, in a total of four unrelated families (Figures 1, S1, and S2; Table 1).

**Table 1:** Mutations identified by custom capture sequencing

Fam	Gene	Details of mutation							Population databases			Del_Pd	CADD score
		Chr	Location	RA	VA	Mut	TC	Protein change	EVS_D	gnomAD_D	1000_G		
1	*RARB*	3	25542776	G	A	AD, Het, NS	Exon 3; c.431G>A	p.Arg144Gln	NR	NR	NR	PP2: (0.998) PbD	27
2	*BMP7*	20	55803304	G	A	AD, Het, NS	Exon 2; c.592C>T	p.Leu198Phe	NR	Hets: 9	NR	PP2: (0.98) PbD	26.7
3	*TFAP2A*	6	10400777	G	GG	AD, Het, FS	Exon 6; c.917_918C>CC	–	NR	NR	NR	–	36
4	*CHD7*	8	61769437	C	A	DeNv, Het, SG	Exon 34; c.7598C>A	p.Ser2533X	NR	NR	NR	–	44

*Note*: Family 1: G>A missense mutation at Chr3: 25542776 (NM_001290216.2) of *RARB* causes c.431 G>A and p.Arg144Gln change. Family 2: G>A missense mutation at Chr20: 55803304 (NM_001719.2) of *BMP7* causes c.592 C>T and p.Leu198Phe change. Family 3: G>GG frameshift mutation at Chr6: 10400777 (NM_003220.2) of *TFAP2A* causes c.917_918C>CC change. Family 4: C>A nonsense mutation at Chr8: 61769437 (NM_017780.3) of *CHD7* causes c.7598 C>A and p.Ser2533X change.

Reference Sequences: *RARB*: NM_001290216.2, NP_001277145.1; *BMP7*: NM_001719.2, NP_001710.1; *TFAP2A*: NM_003220.2; *CHD7*: NM_017780.3, NP_060250.2.

Abbreviations: 1000_G, 1000 genomes; AD, autosomal dominant; Chr, chromosome; Del_Pd, deleteriousness prediction; DeNv, de novo; EVS_D, EVS database; Fam, family; FS, frameshift substitution; gnomAD_D, gnomAD database; Het, heterozygous; Hets, heterozygotes; Mut, type of mutation; NR, not reported; NS, nonsynonymous; PbD, probably damaging; PP2, PolyPhen2 score; RA, reference allele; SG, stop‐gain; TC, transcript change; VA, variant allele.

### 3.2 Clinical findings

*Family 1*: *RARB* autosomal dominant missense mutation causing Arg144Gln protein change (NM_001290216.2, NP_001277145.1; Table 1). The proband was a 38‐year‐old male with bilateral coloboma inherited from the mother, indicating complete penetrance (Figure 1). The right retina showed a small inferior coloboma directly beneath the optic nerve, approximately 1 disc diameter with a circular area of RPE changed temporally. The left retina showed a large inferior coloboma. The proband also revealed a nonspecific color vision deficit and mild phacodynesis on the left. In addition, he had a mild asymmetric sensorineural hearing loss of unclear etiology and severe reflux resulting in Barrett's esophagus. The mother of the proband was a 61‐year‐old woman with bilateral uveal colobomas, mature cataract and sensory exotropia to the left eye, blepharitis, left microcornea, and likely microphthalmia. The left eye showed posterior coloboma with vitreous syneresis. The right eye showed a large chorioretinal coloboma inferior to the disc. The father of the proband was a 64‐year‐old male with no significant clinical findings on a complete ophthalmic exam.

*Family 2*: A heterozygous variant predicted to cause a Leu198Phe change in *BMP7* cosegregated with the coloboma phenotype in mother and daughter (NM_001719.2, NP_001710.1; Table 1 and Figure 1). The proband was a 1‐year‐old girl with unilateral, apparently isolated coloboma of the left eye involving the optic nerve and the inferior retina focally indicating variable expressivity. The left eye revealed mild microcornea and microphthalmia. The mother of the proband revealed a microform of coloboma along with spina bifida occulta at C6 and S1 vertebrae and was designated as affected at the time of the exam, while the father had no evidence of coloboma. Although in silico tools predict this variant to be deleterious, nine heterozygotes are present in the gnomAD database, likely too frequent to cause fully penetrant uveal coloboma. As such, we classified this as a variant of unknown significance and did not pursue it further.

*Family 3*: *TFAP2A* frameshift substitution inherited as autosomal dominant heterozygous mutation (NM_003220.2; Table 1). The proband was an 11‐year‐old male with bilateral coloboma and a history of urinary urgency, left conductive hearing loss likely due to the ruptured tympanic membrane, a possible occipital bone dermoid, and overall clinical finding reminiscent of branchio‐oculo‐facial syndrome (Figure 1). The mother of the proband was a 41‐year‐old female with no evidence of ocular coloboma; however, she showed other clinical findings of branchio‐oculo‐facial syndrome, indicating variable expressivity. The father and the sister of the proband had normal ocular findings.

*Family 4*: A *CHD7* de novo heterozygous mutation causing a stop‐gain (exon 34, NM_017780.3, NP_060250.2; c.7598C>A; p.Ser2533X; Table 1) was identified. The proband was a 1‐year‐old male with bilateral coloboma, right esotropia, left eye preference, and right eye microphthalmia (Figure 1). The right eye revealed a large chorioretinal coloboma affecting the nerve and likely the macula and fovea. The left eye showed circumscribed chorioretinal coloboma around the nerve with areas of retinal dysplasia and pigmentation temporally. The proband presented with other mild features of CHARGE syndrome, such as micropenis, minor dysmorphic features, borderline small kidneys, and mild vertebral differences. The parents of the proband were clinically normal with no evidence of coloboma or other features of CHARGE syndrome.

### 3.3 Mutation in RARB DNA‐binding domain results in decreased protein levels and affects nuclear localization and transcriptional activity

**Figure 2:**
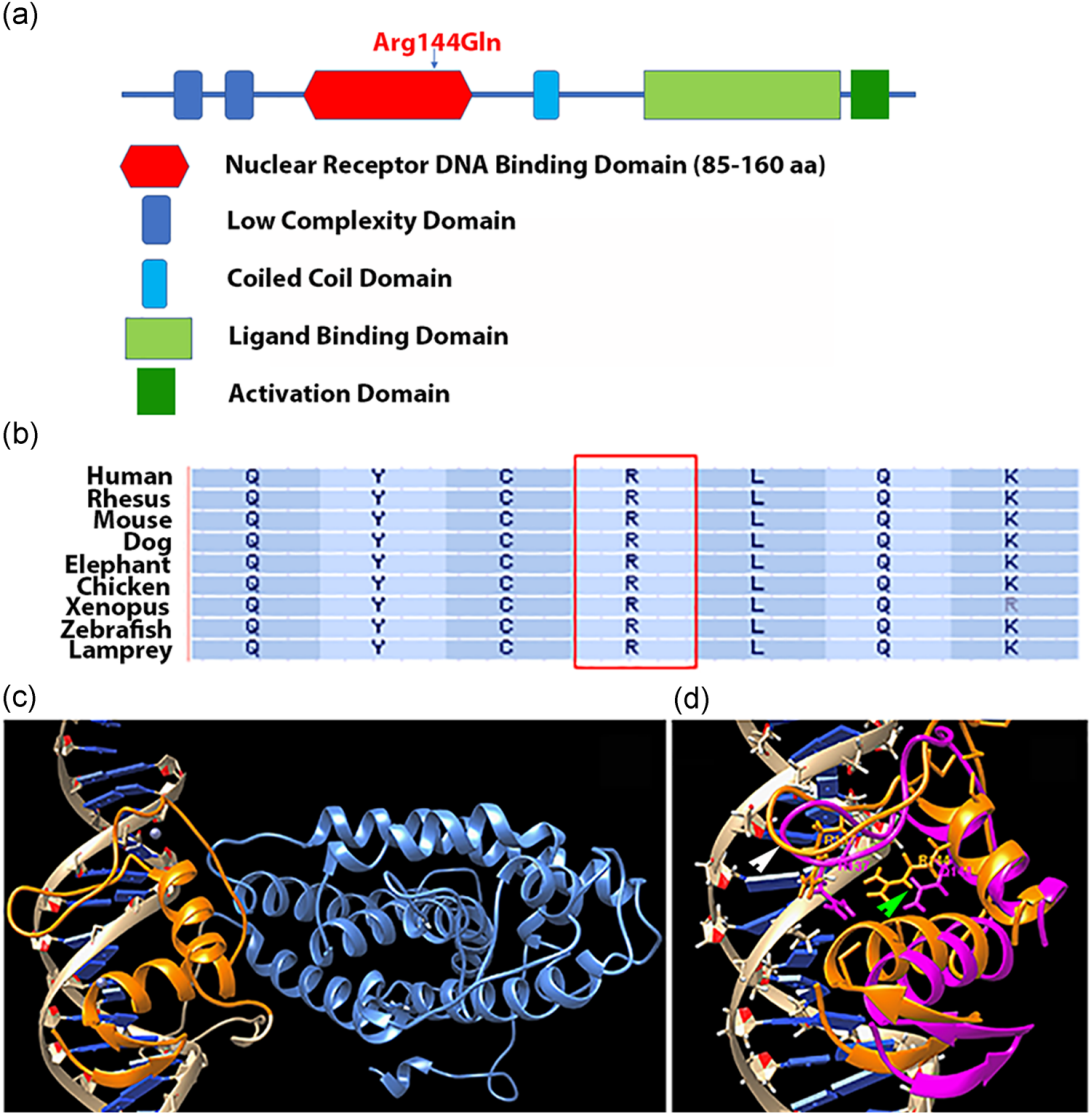
Arg144Gln mutation in RARB affects the binding ability of the DNA‐binding domain. (a) RARB protein with six domains and the approximate location of the Arg144Gln mutation in the DNA‐binding domain. (b) Conservation of the mutated amino acid in the DNA‐binding domain across vertebrate species. (c) Molecular modeling of the interaction of the DNA‐binding domain (orange) with the DNA (beige). The rest of the RARB protein is shown in light blue. (d) Structural superimposition of the two DNA‐binding domains from wild‐type protein (orange) and Arg144Gln mutant variant (magenta) shown in the same DNA double helix groove. In the Arg144Gln mutant, the orientation of the α‐helix interacting with the DNA (green arrowhead) is altered. Location of Arg137 indicated by white arrowhead

We focused specifically on the *RARB* mutation for further investigation as this mutation has been recently reported in a patient with syndromic microphthalmia (Nykamp et al., 2017). RARB protein comprises six distinct regions: two major domains (Figure 2a), a DNA‐binding domain (DBD) and a ligand‐binding domain (LBD) connected by a coiled‐coil hinge region, along with two N‐terminal low complexity domains and a C‐terminal activation domain (Alvarez et al., 2007; Brand et al., 1988; Letunic et al., 2004; Schultz, Milpetz, Bork, & Ponting, 1998). The Arg144Gln mutation is in the DBD, which is a highly conserved region across different vertebrate species from lamprey–zebrafish to rhesus monkey–human (Figure 2a,b and Table S2). On the basis of structural modeling of the wild‐type and mutant protein (Figure 2c,d), the substitution of Arg144 with Gln in the DBD of RARB caused a change in the orientation of a 3‐carbon aliphatic straight chain of Arg137, the distal end of which is capped by a guanidium group. This change is predicted to alter the location of the α‐helix interacting with the DNA in the mutant and it is expected to decrease the DNA‐binding strength of the domain. Protein stability was predicted in silico using the FoldX software (Schymkowitz et al., 2005). The Arg144Gln mutation was predicted to result in improved protein stability by −1.19 kcal/mol compared with the native protein, which suggests a potential loss of native interaction within RARB protein. In addition, the interaction energy between the protein and DNA was predicted to increase by +0.024 kcal/mol, suggesting a complete loss of interaction and thus instability of the protein–DNA complex.

**Figure 3:**
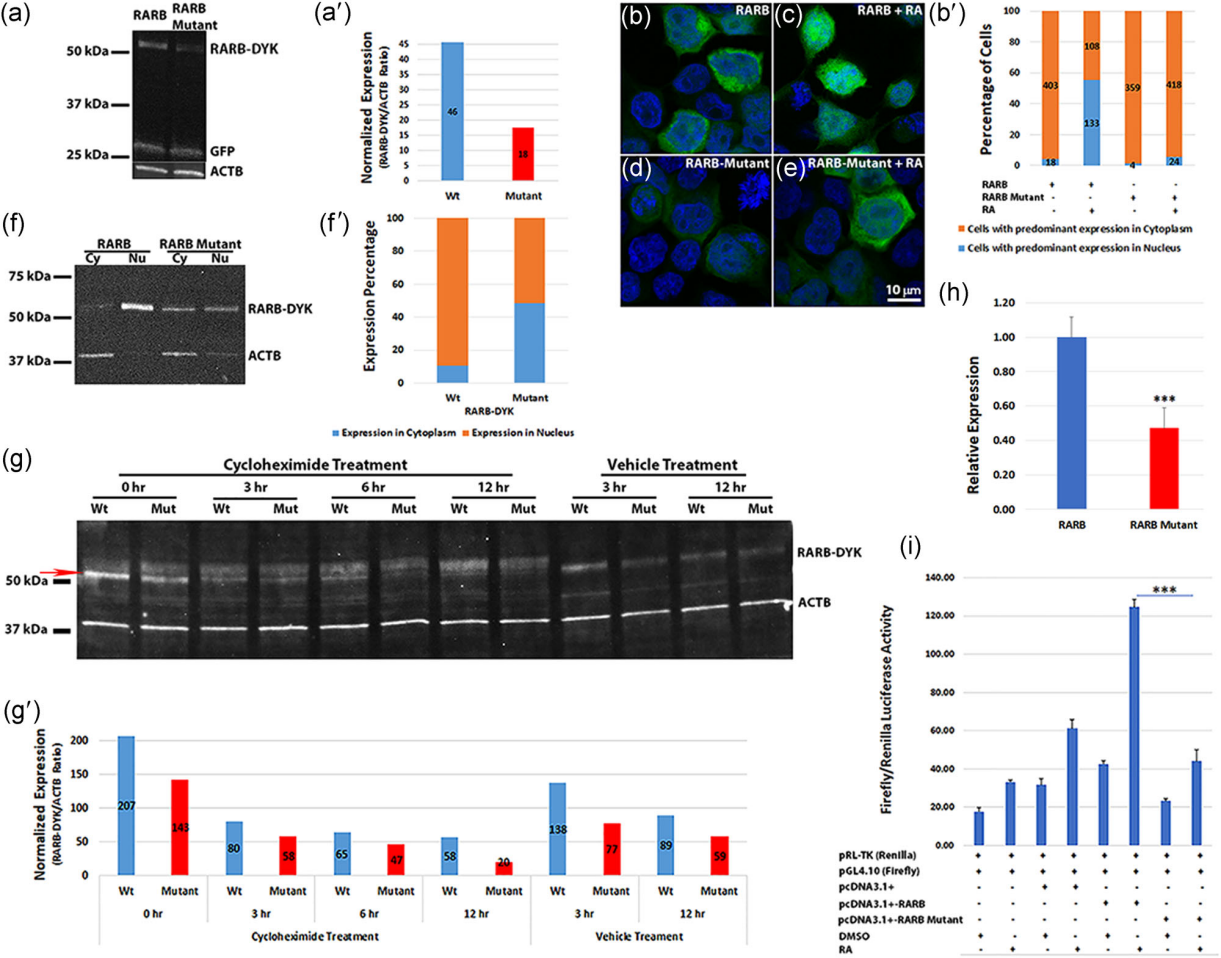
Arg144Gln mutation in RARB results in reduced steady‐state exogenous protein levels, retention in the cytoplasm and affects transcriptional activity. (a) Western blot showing reduced DYK‐tagged RARB‐mutant compared with RARB wild‐type protein in HEK 293‐transfected cells treated with retinoic acid (RA). GFP was used as a control for transfection efficiency and ACTB as a loading control. DYK‐tagged constructs were used for transfection and proteins were detected by DYK, GFP, and ACTB antibodies. (a′) Densitometric analysis of the RARB–DYK bands with normalization to the ACTB. (b–e) Immunofluorescence in HEK 293 cells transfected with wild‐type or mutant *RARB–GFP* expression constructs followed by RA treatment. Both RARB–GFP and RARB‐mutant‐GFP proteins localized to the cytoplasm in the absence of RA. Upon treatment with RA, wild‐type RARB protein localized to the nucleus, while RARB‐mutant protein was mostly retained in the cytoplasm as revealed by the GFP tag. Scale bar in (e) applies to all images. (b′) Bar graph of the percentage of cells showing predominant localization of the transfected protein in the cytoplasm or the nucleus. Values on the graph indicate the total number of cells counted. Only immunolabeled cells were counted from five different fields. Cells with predominant localization in the cytoplasm (brown); cells with predominant localization in the nucleus (blue). With RA treatment, RARB wild‐type protein showed significantly more nuclear localization compared with the RARB‐mutant protein (*p* < .01). (f) Nuclear and cytoplasmic fractions separated from transfected HEK 293 cells treated with RA. Wild‐type RARB protein was mostly localized to the nucleus, while RARB‐mutant protein was observed both in the nucleus and in the cytoplasm. (f′) Percentage expression in the nucleus and cytoplasm of RARB–DYK bands from the densitometric analysis. The mutant protein showed reduced expression and localization to the nucleus compared with the wild‐type protein. (g) Protein degradation assay. A *T*_0_ timepoint was harvested before cycloheximide addition (0 hr). The mutant protein was barely visible after 12 hr of cycloheximide treatment, whereas, wild‐type RARB protein was still detected. The red arrow indicates the RARB–DYK band used for densitometric analysis. (g′) Densitometric analysis of the RARB–DYK bands normalized to ACTB. (h) qRT‐PCR of wild‐type and mutant *RARB* transcripts from the transfected HEK 293 cells normalized to *GAPDH* and to the neomycin‐resistance gene from the expression vector construct. Expression of the *RARB*‐mutant transcript was significantly reduced compared with the wild‐type transcript. ***p < .01. (i) Transcriptional activity of RARB wild‐type and mutant proteins. Transcriptional activity is expressed as Firefly/Renilla luciferase activity ratio. RA treatment induced a significant increase in the transcriptional activity of wild‐type RARB but not of RARB‐mutant, ***p < .001. GFP, green fluorescent protein; HEK 293, human embryonic kidney 293; qRT‐PCR, quantitative real‐time polymerase chain reaction

Because mutations in the DBD may also affect the nuclear localization of a protein (Bunn et al., 2001), we investigated cellular localization and transcriptional activity of RARB *in vitro*. We predicted that the mutant protein would respond to RA treatment like wild‐type as the LBD appeared unaffected by the mutation. We transfected HEK 293 cells in culture with wild‐type and mutant *RARB* expression constructs, followed by RA treatment. By western blot analysis, cells transfected with the wild‐type *RARB* expression construct showed higher steady‐state levels of exogenous RARB protein compared with cells transfected with *RARB*‐mutant expression construct (Figures 3a and 3a′). Immunofluorescence showed that most of the RARB‐mutant protein was retained in the cytoplasm while the wild‐type protein was mostly detected in the nucleus (Figures 3b–e and 3b′). We obtained comparable results when cells were cultured in charcoal‐stripped serum‐containing medium or in regular serum‐containing medium and when transfected with either DYK‐tagged or GFP‐tagged expression constructs (Figure S3). We confirmed the localization of mutant and wild‐type RARB proteins by separating the nuclear and cytoplasmic fractions and showed that the mutant protein was equally distributed between the cytoplasm and the nucleus whereas the highest proportion of wild‐type protein was present in the nuclear fraction in the presence of ligand (Figures 3f and 3f′). Translation blocking with cycloheximide treatment of transfected cells in culture confirmed the reduced expression of the mutant protein at *T*_0_ and complete degradation over a period of 12 hr compared to only partial degradation of the wild‐type protein over the same time period (Figures 3g and 3g′). Gene expression quantification with qRT‐PCR revealed significantly less mutant *RARB* transcripts compared with the wild‐type (Figure 3h). Taken together, these results suggest that the reduced steady‐state levels of mutant RARB are likely due to a combination of reduction in both mRNA and protein stability.

We then tested the transcriptional activity of the wild‐type and mutant RARB proteins using a dual luciferase assay and the ratio of Firefly/Renilla luciferase intensity. The promoter region of the human *CYP26A1* gene that contains the binding sites for RARB protein was PCR amplified and cloned into the pGL4.10 (luc2) vector to study the response to RA treatment. Wild‐type RARB transcriptional activity was higher than baseline and control empty vector and was greatly increased by RA treatment. On the contrary, transcriptional activity of the RARB‐mutant protein was reduced at baseline and only slightly increased upon RA treatment (Figure 3i). Transcriptional activity of the RARB‐mutant protein was not significantly different from that of the control empty vector.

### 3.4 RARB‐mutant protein does not alter the localization of RXR proteins

**Figure 4:**
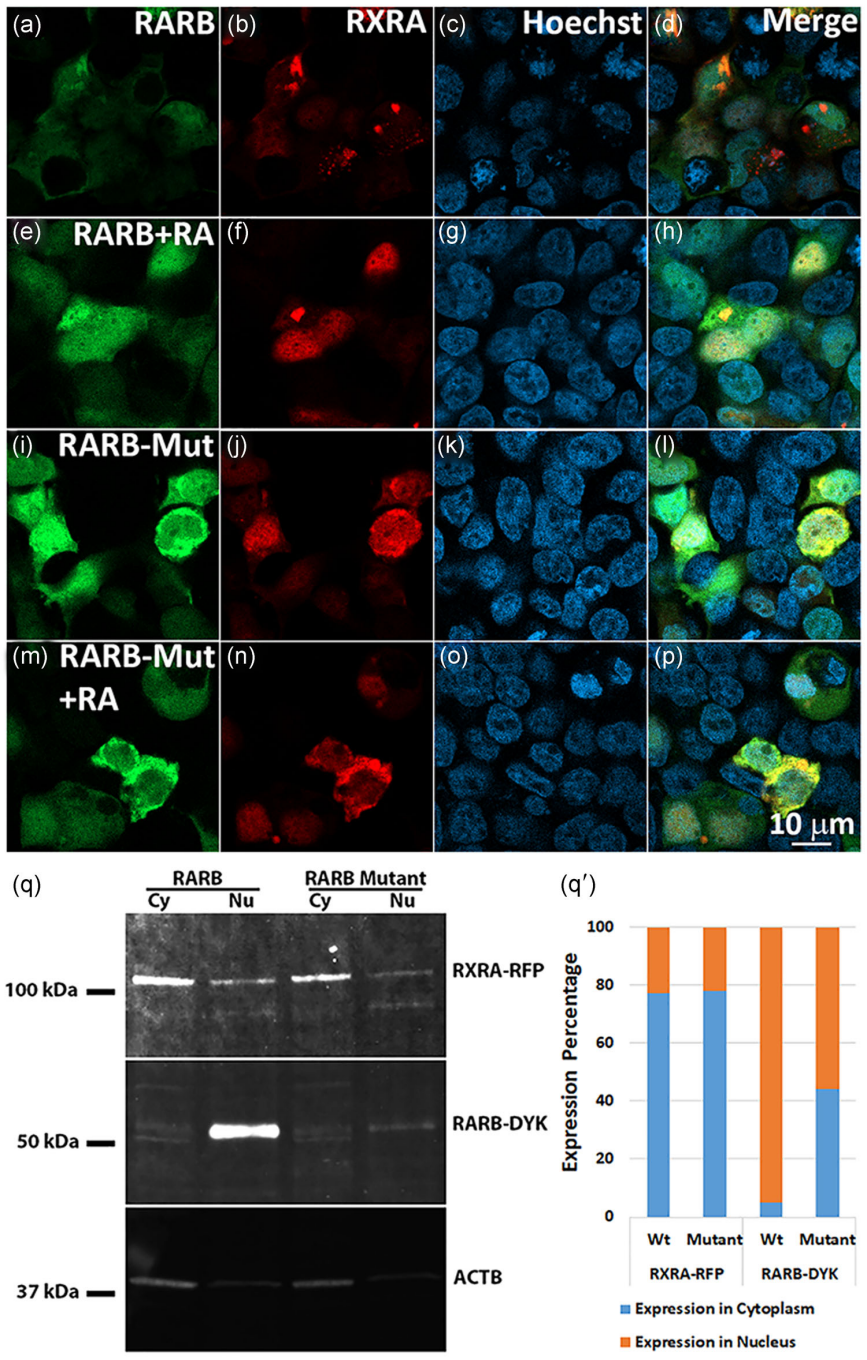
RARB‐mutant protein does not alter the localization of RXR proteins. (a–p) Immunofluorescence of HEK 293 cells treated with DMSO/RA following transfection with GFP‐tagged wild‐type *RARB* or *RARB*‐mutant and RFP‐tagged *RXRA* expression constructs*.* Wild‐type RARB along with RXRA protein appeared to translocate to the nucleus upon RA treatment whereas partial retention of RXRA protein was seen in the cytoplasm along with the RARB‐mutant protein as revealed by colocalization of GFP and RFP signals from the fusion proteins. Scale bar in (p) applies to all images. (q) Western blots of protein lysates from cytoplasmic and nuclear fractions separated from cells cotransfected with wild‐type or mutant *RARB–DYK* and *RXRA–RFP* expression constructs and treated with RA. RXRA localization appeared not affected in cells cotransfected either with *RARB* wild‐type or mutant expression constructs. However, RARB wild‐type protein was mostly localized to the nucleus while the RARB‐mutant protein was mostly localized to the cytoplasm. (q′) Expression percentage of the RARB–DYK and RXRA–RFP bands from the densitometric analysis. The mutant RARB protein showed reduced expression and translocation to the nucleus compared with the wild‐type protein while the RXRA showed no difference in localization. DMSO, dimethyl sulfoxide; GFP, green fluorescent protein; HEK 293, human embryonic kidney 293; RA, retinoic acid

RA receptors have been shown to form heterodimers with RXRs to transduce the RA signal (Chandra et al., 2017; Glass, 1996; Mangelsdorf & Evans, 1995; Mangelsdorf et al., 1995). We hypothesized that the fraction of RARB‐mutant protein retained in the cytoplasm heterodimerizes with other RXRs and prevents them from translocating into the nucleus. We performed cotransfection of GFP‐tagged *RARB* wild‐type or mutant plasmid constructs with RFP‐tagged *RXRA* or *RXRG* plasmid constructs in HEK 293 cells. Colocalization of GFP and RFP fluorescence suggested predominant localization of RXRA protein in the nucleus when cotransfected with wild‐type *RARB* (Figure 4a–h) while it was partially retained in the cytoplasm of cells cotransfected with the *RARB*‐mutant construct in the presence of RA (Figure 4i–p). Cellular fractionation studies confirmed the predominant localization of the wild‐type RARB protein to the nucleus while the mutant RARB protein was mostly retained in the cytoplasm. However, fractionation did not reveal any difference in the localization of the RXRA–RFP fusion protein when cotransfected either with wild‐type or mutant *RARB* construct (Figures 4q and 4q′). Localization of RXRG protein showed no significant difference when cotransfected either with wild‐type or mutant *RARB* construct (Figure S4a–d).

In summary, the Arg144Gln mutation in *RARB* identified in Family 1 is localized to the DNA‐binding region of the protein and appears to partially interfere with the nuclear localization and transcriptional activity of RARB.

### 3.5 Human *RARB* mRNA partially reduces the ocular phenotype caused by *rarga* morpholino knockdown in zebrafish embryos

**Figure 5:**
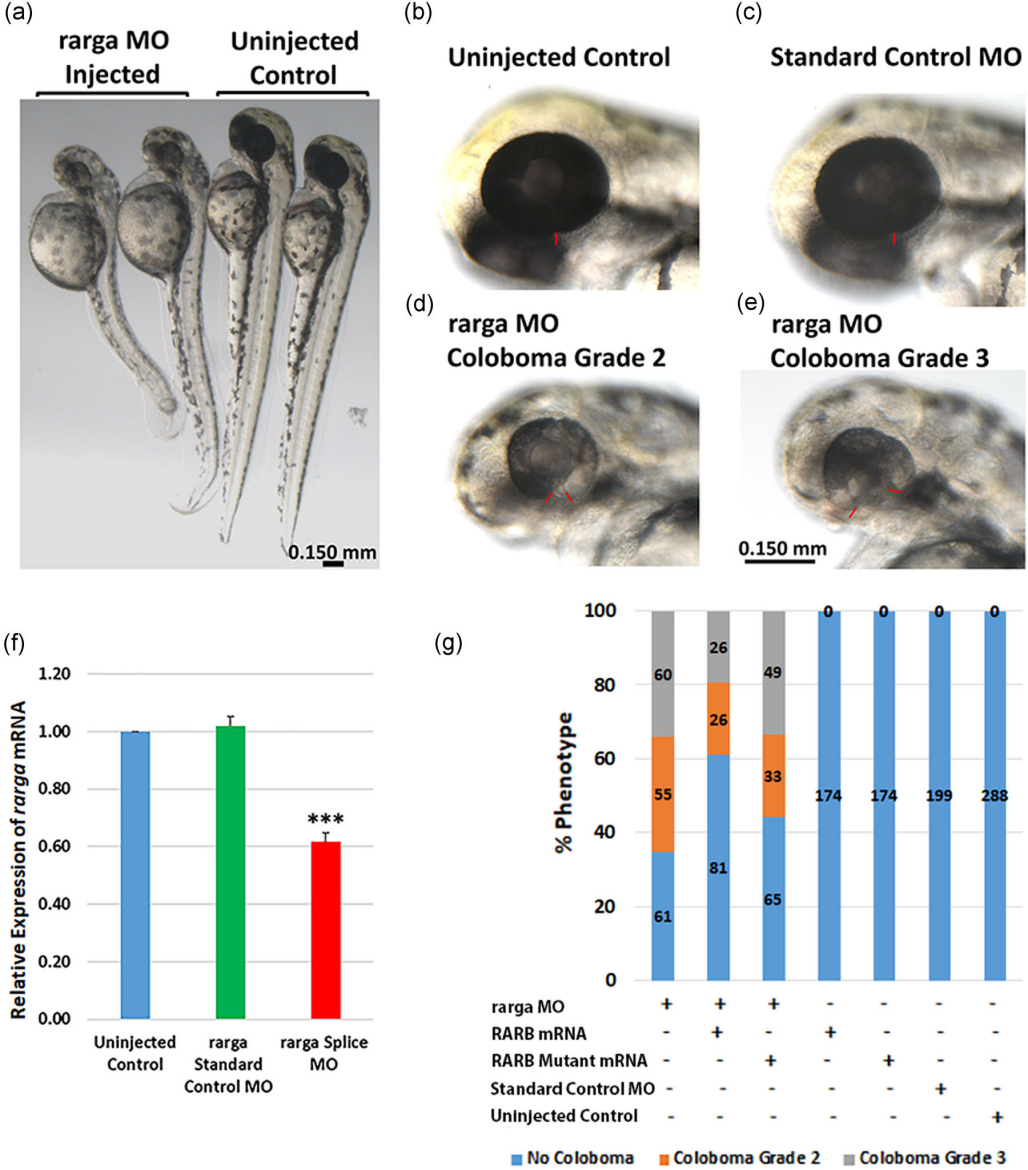
Ocular coloboma phenotype and partial rescue in *rarga* morpholino (MO) knockdown zebrafish embryos. (a) *rarga* MO‐injected embryos show a slightly smaller body and head sizes compared with uninjected zebrafish embryos at 48 hr postfertilization (hpf). (b) Uninjected wild‐type with normal eye. (c) Standard control MO‐injected embryos. Ocular size is comparable to the wild‐type uninjected embryos and both reveal no coloboma in the eye or other phenotypes at 3.75–7.5 ng MO concentrations. (d–e) *rarga* MO‐injected embryos revealed concentration‐dependent ocular coloboma as Grade 2 (narrow, d) or Grade 3 (wide, e) optic fissure closure defects. The red bars indicate the margins of the optic fissure. Scale bar in (e) applies to panels b–e. (f) *rarga* transcript expression by qRT‐PCR was significantly reduced in MO knockdown zebrafish embryos; ***p < .001. (g) Human *RARB* mRNA but not *RARB*‐mutant mRNA partially reduced the coloboma phenotype in *rarga* MO knockdown embryos. Embryos were injected with *rarga* splice‐blocking MO along with either human wild‐type *RARB* or *RARB‐mutant* mRNA and coloboma phenotype was analyzed after 48 hpf. The values on the bars represent the number of embryos injected or analyzed. qRT‐PCR, quantitative real‐time polymerase chain reaction; mRNA, messenger RNA

To understand the role of *RARB* in optic fissure closure, we performed functional studies in the developing zebrafish. The closest homolog to human *RARB* in zebrafish is *rarga*, with 79% identity on phylogenetic analysis (Figure S5). *rarga* is expressed in the cranial mesoderm and periocular mesenchyme and represents one of the most abundantly expressed retinoic receptor genes during the early stages of zebrafish development (Linville, Radtke, Waxman, Yelon, & Schilling, 2009; Oliveira et al., 2013). We first tested if knockdown of *rarga* gene causes coloboma phenotype in zebrafish. We used both translational (TB) and splice‐blocking (SB) morpholinos and a combination of both to knockdown the *rarga* gene by microinjection into 1–2 cell‐stage zebrafish embryos (Table 2). The *rarga* gene knockdown resulted in ocular coloboma with dose‐dependent severity of the phenotype and other systemic phenotypes (Figure 5a–e and Table 2). The expression levels of *rarga* mRNA were significantly reduced in the *rarga* SB morpholino‐injected embryos compared with the uninjected and standard morpholino‐injected control embryos (Figure 5f). The ocular phenotype included microphthalmia, and a range of mild (e.g., Grade 2, Panel 5d) to severe (e.g., Grade 3, Panel 5e) forms of coloboma depending on the dose of morpholino. We then used the human *RARB* wild‐type and mutant mRNA to rescue the ocular phenotype caused by *rarga* gene knockdown. Upon coinjection of *rarga* SB morpholino with *RARB* wild‐type mRNA, 39% of embryos displayed coloboma compared with 65% of embryos injected with morpholino alone. Furthermore, the percent of more severe (Grade 3) coloboma dropped to 19% from 34%. Conversely, 56% of embryos coinjected with *rarga* SB morpholino and the *RARB*‐mutant mRNA showed a coloboma phenotype, 33% of which were Grade 3 (Figure 5g and Table S3). The results were consistently replicated during multiple injections and the direction of change in the proportion of embryos with coloboma suggests an effect of *RARB* wild‐type mRNA but not of *RARB‐*mutant mRNA*.* Thus, knockdown of the zebrafish equivalent to *RARB* gene, *rarga*, results in a phenotype reminiscent of human coloboma.

**Table 2:** *rarga* morpholino injections in zebrafish embryos

MO	Conc^a^ (ng)	No col/grade 1 no. of emb (%)	Mild col/grade 2 No. of emb (%)	Severe col/grade 3 no. of emb (%)	Total emb injected
TB MO	3.75	73 (40)	40 (22)	69 (38)	182
	5	78 (42)	9 (5)	97 (53)	184
	7.5	32 (26)	11 (9)	80 (65)	123
SB MO	3.75	116 (94)	5 (4)	3 (2)	124
	5	31 (65)	10 (21)	7 (15)	48
	7.5	52 (33)	29 (18)	78 (49)	159
TB + SB MO	1.87 + 1.87	47 (62)	13 (17)	16 (21)	76
	2.5 + 1.87	21 (20)	19 (18)	64 (62)	104
Std_C MO	3.75	209 (99)	2 (1)	0 (0)	211
	5	285 (100)	1 (0)	0 (0)	286
	7.5	196 (99)	2 (1)	0 (0)	198
Uninj Emb	–	939 (100)	1 (0)	1 (0)	941

*Note*: Knockdown of *rarga* gene with translation blocking (TB) and splice blocking (SB) and a combination of TB and SB morpholinos resulted in a dose‐dependent severity in ocular coloboma phenotype in zebrafish embryos.

Abbreviations: col, coloboma; Conc, concentration; Emb, embryos; MO, morpholino; ng, nanogram; SB, splice blocking; Std_C, standard control; TB, translation blocking; Uninj, uninjected.

^a^ Concentration‐dependent increase in coloboma severity observed in embryos with both translation‐ and splice‐blocking morpholinos and when injected in combination. Each of the morpholino injections was performed at least three to four times and the total number of embryos injected is included in the table.

## 4 DISCUSSION

Custom capture or targeted gene sequencing has been a cost‐effective approach to detect mutations in genes known to be associated with a specific disease condition (Aparisi et al., 2014; Bonachea et al., 2014; Patel et al., 2018; Rehm, 2013; Shang et al., 2018; Trujillano et al., 2014; X. Wang et al., 2016). Previous studies exploring mutations in coloboma‐associated genes have been limited to a subset of genes in affected families (Gonzalez‐Rodriguez et al., 2010; Williamson & FitzPatrick, 2014). In the present study, we performed targeted sequencing of 37 genes known to be associated with human coloboma in a large cohort of patients with syndromic and nonsyndromic ocular coloboma. We identified novel variants in genes such as *TFAP2A* and *CHD7*, and previously reported variants in *RARB* and *BMP7*, indicating that mutations in known coloboma‐associated genes account for only 4.5% of the coloboma cohort analyzed. Congenital disorders such as coloboma and other conditions were shown to exhibit high genetic heterogeneity and targeted sequencing of disease‐associated genes may yield the limited outcome of potential variants (DaRe, Vasta, Penn, Tran, & Hahn, 2013; Perez Millan et al., 2018; Redin et al., 2014). A recent report on diagnosing genetic developmental eye disorders indicated a low diagnostic yield of 8% for microphthalmia, anophthalmia, and coloboma disease, similar to the yield reported here (Patel, Hayward et al., 2019). Custom capture or targeted next‐generation sequencing arrays (Illumina HiSeq 2000 and 2500) have been widely used for mutation detection of several genetic disease conditions, although, with variable yield depending on the genetic disorder and the precise population studied. Our patient population largely excluded probands where a clear, syndromic diagnosis might be made and molecular testing serves more for confirmation of a clinical diagnosis (e.g., patients who easily meet diagnostic criteria for CHARGE syndrome); had more of such patients been included in this cohort, we posit our yield would have been higher. Our US‐based population was also largely devoid of known consanguinity. In fact, recent studies using next‐generation sequencing panels have reported 39% mutation detection in orodental genetic disorders and 61% mutation detection in a cohort of highly consanguineous individuals with microphthalmia, supporting these hypotheses (Patel et al., 2018; Prasad et al., 2016). Although we did not validate our custom capture sequencing panel with known mutations, we considered only the variants with an MPG score >10 and an MPG score to a depth of coverage ratio >0.5 indicating a high‐quality genotype call for the variants selected. All the variants thus identified were evident in Sanger sequencing indicating the efficiency of the sequencing panel. Of the four variants identified, we further investigated the cellular and molecular pathways affected by the Arg144Gln mutation in RARB and partially rescued the developmental phenotype in zebrafish.

The DBD is highly conserved among all RAR and RXR receptors and contains two zinc finger motifs essential for binding to the DNA (Alvarez et al., 2007; Swift, Hays, & Petty, 2008). The Arg144Gln mutation we identified is located in the putative zinc finger motif of the DBD of RARB. Protein modeling predicted the mutation to cause structural changes that could render the DNA‐protein complex less stable. Previous studies have shown that RA upregulates *RARB* during cellular proliferation (H. X. Liu, Ly, Hu, & Wan, 2014). Consistent with these reports, cells treated with RA following transfection with the wild‐type *RARB* expression construct showed increased protein levels when compared with cells transfected with mutant *RARB.* Studies on synthetic mutations in the DBD of thyroid nuclear receptors, that have a similar structure and functional domains, revealed impaired nuclear localization of the receptors (Bunn et al., 2001). Consistent with these findings, the nuclear translocation of mutant RARB upon RA stimulation was only partial. Two nuclear localization signals can be identified in nuclear receptors, one in the N‐terminal region and the other in the hinge region (Mavinakere, Powers, Subramanian, Roggero, & Allison, 2012; J. Zhang, Roggero, & Allison, 2018). The Arg144Gln mutation in RARB is located at the end of the DBD and we predicted that structural changes introduced by this mutation may alter the orientation of the adjacent hinge region thereby affecting nuclear localization of the protein. Partial translocation to the nucleus and impaired DNA‐binding capability explain the loss of transcriptional activity of RARB‐mutant compared with wild‐type RARB.

The previously reported mutation in the DBD of RARB (Arg119*) caused a truncation and total loss of function (Srour et al., 2013). However, the Arg144Gln mutant described in this study is a viable protein that is mostly retained in the cytoplasm. We hypothesized that mutant RARB that had lost its ability to translocate to the nucleus would “trap” its heterodimeric partners in the cytoplasm. RA and other nuclear receptors such as thyroid hormone and vitamin D3 exert their function by heterodimerizing with RXRs (Germain & Bourguet, 2013; Khorasanizadeh & Rastinejad, 2001). While ligands (at‐RA for RARs and cis‐RA for RXRs) are important for translocation to the nucleus, RAR and RXR receptors have also been shown to translocate to the nucleus independently of the presence of the ligand and their heterodimerization was shown to play a role in nuclear localization (Baumann, Maruvada, Hager, & Yen, 2001; Mavinakere et al., 2012). RARB*/*RXRA and RARG*/*RXRA heterodimers are instrumental for ocular morphogenesis and RXRA is the main RXR dimerizing with RA receptors during development (Kastner et al., 1997; Krezel et al., 1996; Mark, Ghyselinck, & Chambon, 2006; Mascrez et al., 2001). By confocal imaging, it appeared that RXRA protein was retained in the cytoplasm when cotransfected with *RARB*‐mutant but not with wild‐type *RARB*. However, cellular fractionation and quantification did not reveal any difference in RXRA–RFP protein nuclear localization when cotransfected with either wild‐type or mutant *RARB*. Therefore, RARB‐mutant protein does not seem to affect the localization of at least two members of the RXR family.

The clinical manifestation in the proband and the mother carrying the Arg144Gln RARB mutation were mainly bilateral uveal coloboma. These findings represent a system‐restricted phenotype compared with previous reports of *RARB* mutations in humans with either total loss‐of‐function or twofold to threefold gain‐of‐function showing involvement of several organs, including eye, heart, diaphragm, lungs, brain, and locomotor system (Srour et al., 2013, 2016). Srour et al., showed that stop‐gain mutation in the DBD and frameshift mutation in LBD caused a total loss‐of‐function while missense mutations in the LBD resulted in gain‐of‐function with all the affected showing multisystemic phenotypes. Taken together, these reports and our findings indicate that the RARB protein levels are critical for normal organogenesis and RA‐sensitive organ systems such as the eye are highly vulnerable to subtle changes in RARB levels. The zebrafish homolog of the human *RARB* gene, *rarga*, is expressed in the cranial mesoderm and periocular mesenchyme and is reported to be the most abundant RA receptor expressed during early stages of zebrafish embryonic development (Linville et al., 2009; Oliveira et al., 2013). Previous reports in zebrafish indicate that RA receptor signaling is required for morphogenesis of the ventral optic cup and closure of the optic fissure (Lupo et al., 2011). We reasoned that by knocking down *rarga* we could recapitulate coloboma in zebrafish and use this as a developmental system to attempt to rescue the phenotype. Indeed, morpholino knockdown of *rarga* caused dose‐dependent ocular coloboma; a comparatively mild and partial rescue of the phenotype was observed only with coinjection of human wild‐type *RARB* mRNA. We posit that the rescue would have been more robust had there been an equivalent of *RARB* in zebrafish. Nonetheless, this trend was reproducible in three separate experiments, as was our observation that the mutant *RARB* mRNA did not rescue as well.

This study was initiated with the goal to apply custom capture targeted sequencing for the identification of potential disease‐causing variants in known coloboma‐associated genes. The custom capture screening contributed to the identification of mutations in some of the syndromic cases, while the nonsyndromic cases remain elusive. Because of the high genetic heterogeneity, we conclude that broader sequencing approaches, such as whole‐exome or genome sequencing, will be required for gene discovery in this disease. The mutation we identified in the DBD of RARB prompted us to further investigate its consequences at the cellular and molecular level. Our data suggest that RARB plays an essential role in RA signaling during optic fissure closure in eye development. Further work on RARB and its downstream targets may help elucidate the RA‐induced transcriptional network involved in optic fissure closure.

## CONFLICT OF INTERESTS

The authors declare that there are no conflict of interests.

## Supporting information

Supporting information

## Data Availability

The variants reported in this study have been submitted to dbSNP public database. (URL: https://www.ncbi.nlm.nih.gov/projects/SNP/).
