# Supplementary material for: High‐throughput custom capture sequencing identifies novel mutations in coloboma‐associated genes: Mutation in DNA‐binding domain of retinoic acid receptor beta affects nuclear localization causing ocular coloboma
Source: Hum Mutat. 2019 Dec 9;41(3):678–95. doi: 10.1002/humu.23954 (PMC7027867; doi:10.1002/humu.23954)
Supplement: Supplementary file 1 — Supporting information [file HUMU-41-678-s001.pdf]

Supp_Mat:

Supp. Figure S1

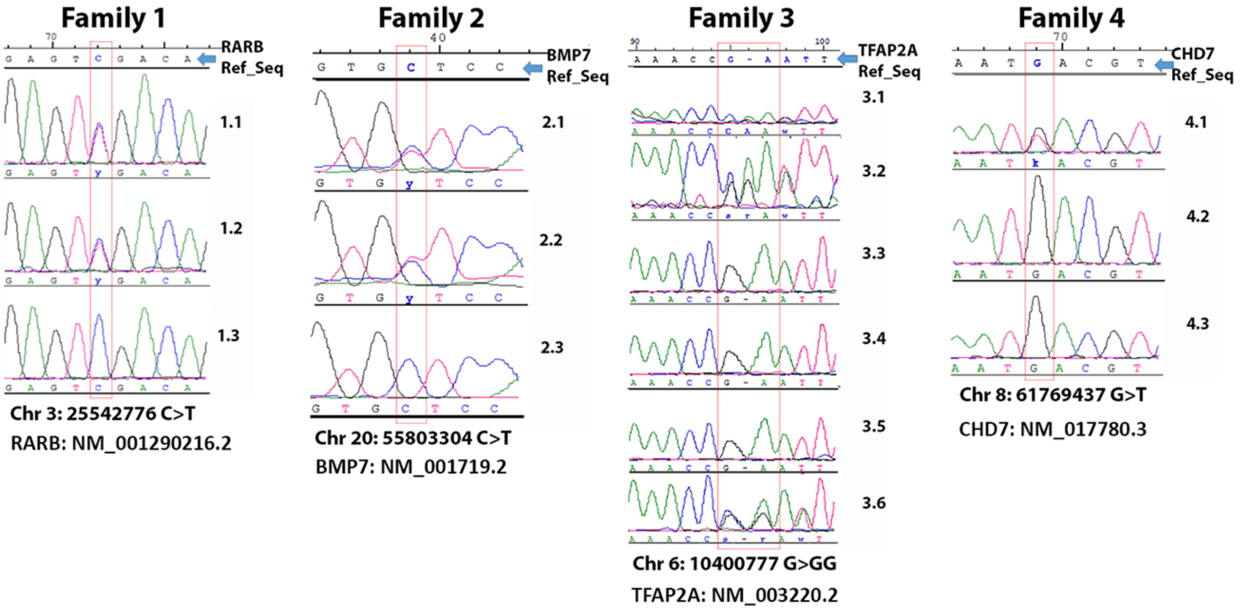

Supp. Figure S1: Chromatograms reveal segregation of the mutation to the affected individuals. Family 1: G>A (sequence in reverse direction shown as C>T) heterozygous missense change in *RARB* at Chr3: 25542776 in the male proband (1.1) and the mother (1.2) (NM_001290216.2, NP_001277145.1). Family 2: G>A (sequence in reverse direction shown as C>T) heterozygous missense change in *BMP7* at Chr20: 55803304 in the female proband (2.1) and the mother (2.2) (NM_001719.2, NP_001710.1). Family 3: G>GG frameshift change in *TFAP2A* at Chr6: 10400777 in the male proband (3.1), his half-sibling (3.6) and the mother (3.2) (NM_003220.2). Family 4: C>A (sequence in reverse direction shown as G>T) heterozygous nonsense change in *CHD7* at Chr8: 61769437 in the male proband (4.1) (NM_017780.3, NP_060250.2).

Supp. Figure S2

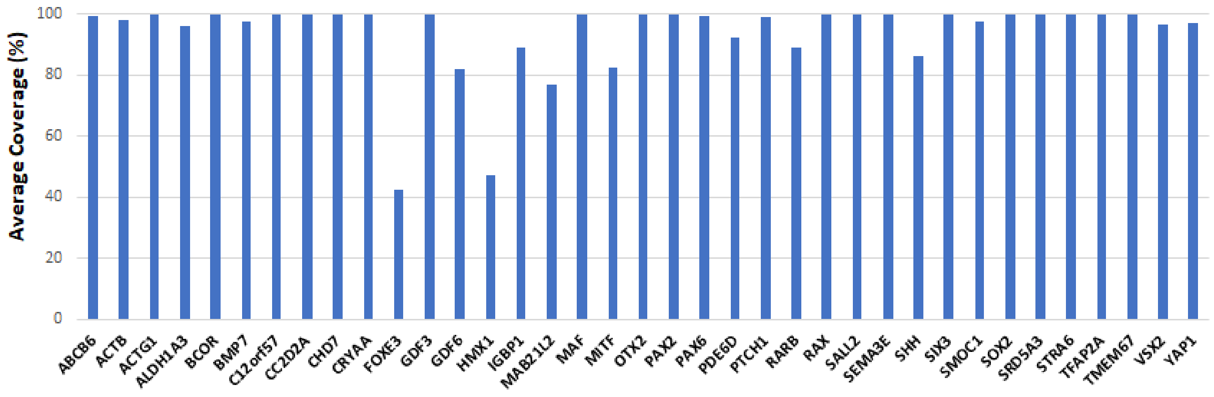

Supp. Figure S2: Custom capture sequencing of coloboma-associated genes. Sequencing of the exonic regions reveals about 90% coverage (i.e., sites within the targets that had MPG score greater than 10 and an MPG score to depth of coverage ratio greater than 0.5) in most of the coloboma-associated genes.

Supp. Figure S3

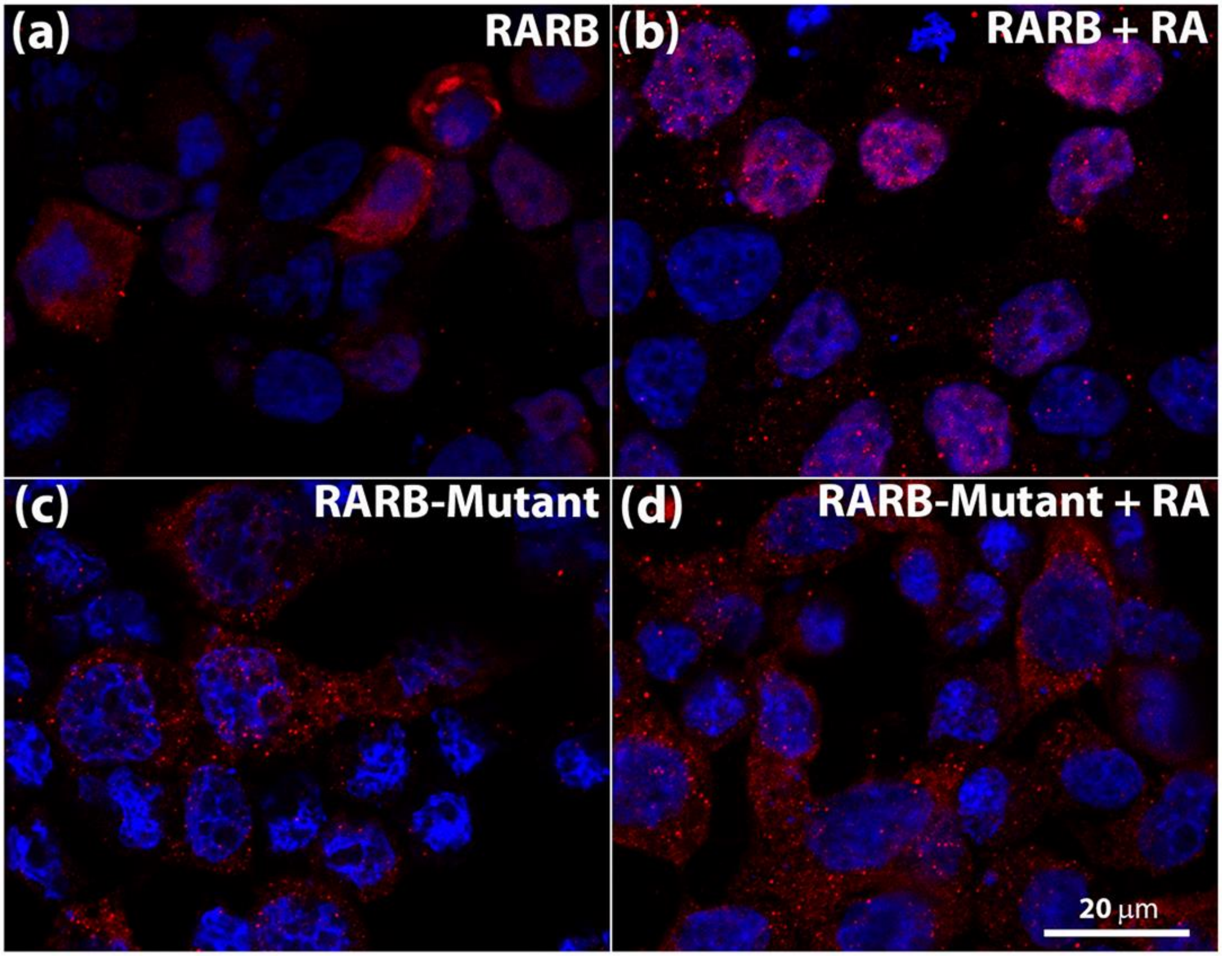

Supp. Figure S3: Immunofluorescence in HEK 293 cells transfected with wild type *RARB-DYK* or *RARB*-mutant-*DYK* expression constructs followed by RA treatment. (a, c) Both RARB and RARB-mutant proteins localized to the cytoplasm in the absence of RA. (b, d) Upon treatment with RA, wild type RARB protein localized to the nucleus (b), while RARB-mutant protein was mostly retained in the cytoplasm (d). Scale bar in (d) applies to all panels.

Supp. Figure S4

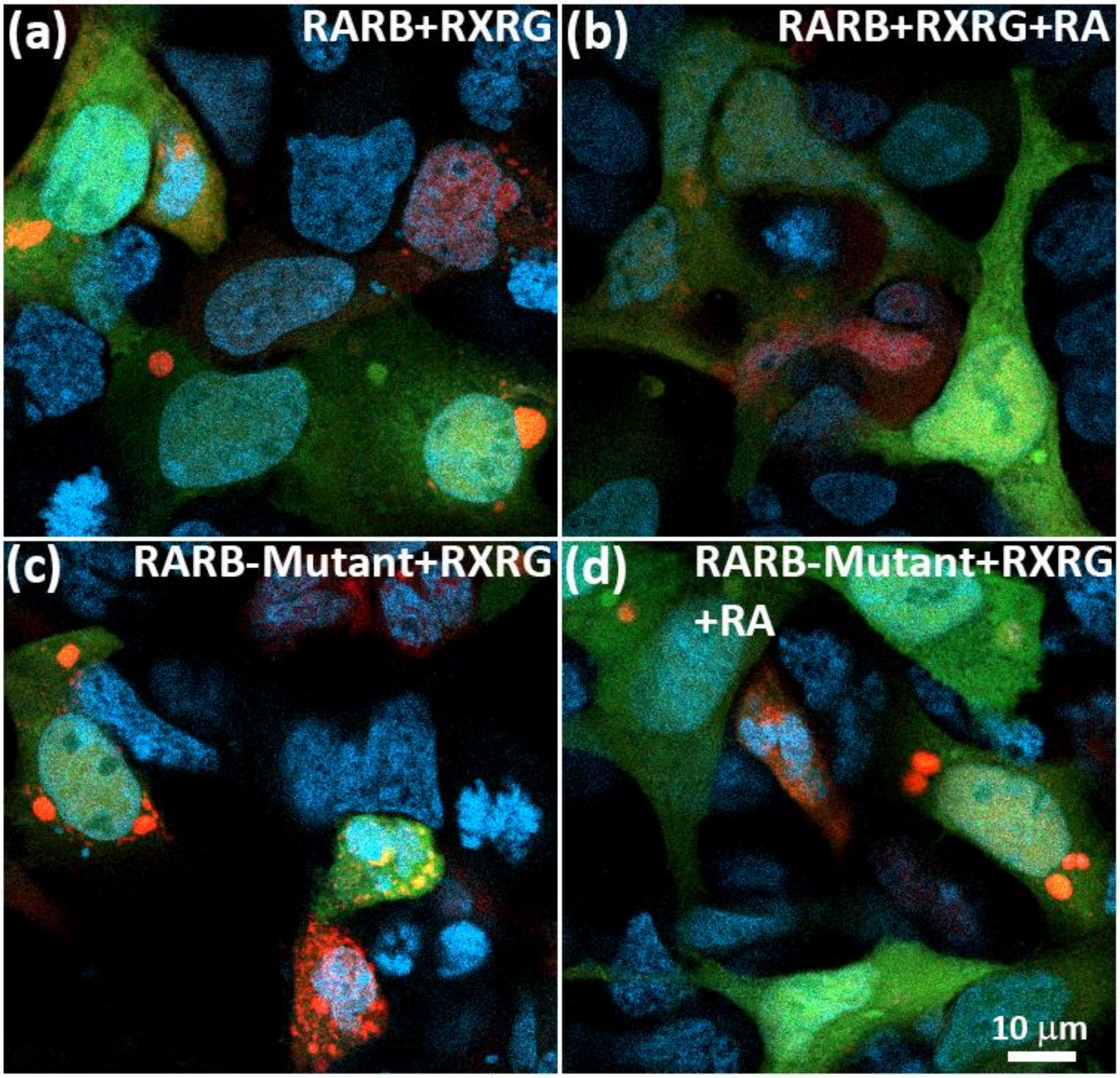

Supp. Figure S4: Immunofluorescence in HEK 293 cells co-transfected with wild type RARB-GFP or RARB-mutant-GFP and RXRG-RFP expression constructs followed by RA treatment. (a, c) RARB-GFP and RARB-mutant-GFP along with RXRG-RFP proteins are more localized to the cytoplasm in the absence of RA. (b, d) Upon treatment with RA, no significant difference in the translocation of RXRG-RFP was observed between cells co-transfected with wild type or mutant *RARB-GFP* constructs.

Supp. Figure S5

(a)

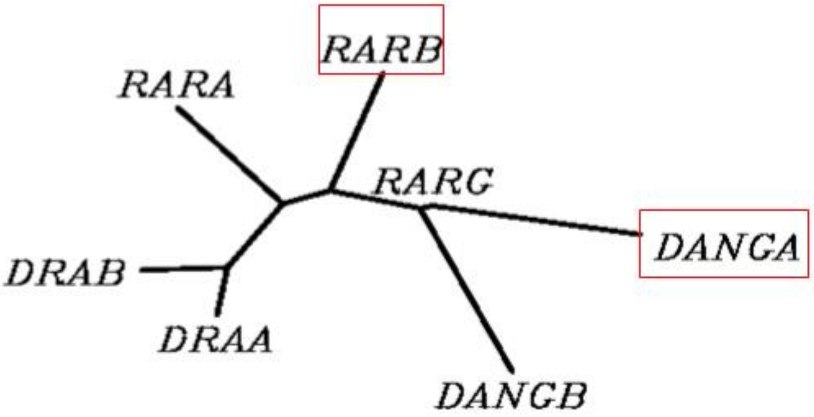

(b)

Score	Expect	Method	Identities	Positives	Gaps
629 bits(1623)	0.0	Compositional matrix adjust.	326/415(79%)	363/415(87%)	5/415(1%)

```
Query  1    MFDCMDVLSVSPGQILDFYTASPSSCMLQEKALKACFSGLTQTEWQHRHTAqsietqsts  60
            MFDCM+ L + P Q+ D    +  +CML++ +    ++GL    W    + +S+ETQSTS
Sbjct  1    MFDCMEALGMGPRQLYD--VTNRGACMLRKAS--PFYAGLDPFAWTGTASVRSVETQSTS  56

Query  61   seelvpsppsplpppRVYKPCFVCQDKSSGYHYGVSACEGCKGFFRRSIQKNMIYTCHRD  120
            SEE+VPS PSP PPPRVYKPCFVCQDKSSGYHYGVS+CEGCKGFFRRSIQKNM+YTCHRD
Sbjct  57   SEEMVPSSPSPPPPPRVYKPCFVCQDKSSGYHYGVSSCEGCKGFFRRSIQKNMVYTCHRD  116

Query  121  KNCVINKVTRNRCQYCRLQKCFEVGMSKESVRNDRNKKKKETSKQECT-ESYEMTAELDD  179
            KNC INKVTRNRCQYCRLQKCFEVGMSKE+VRNDRNKKKK+   +    ESYE++ EL++
Sbjct  117  KNCQINKVTRNRCQYCRLQKCFEVGMSKEAVRNDRNKKKKDVKDEVIPPESYELSGELEE  176

Query  180  LTEKIRKAHQETFPSLCQLGKYTTNSSADHRVRLDLGLWDKFSELATKCIIKIVEFAKRL  239
            L  K+ KAHQETFPSLCQLGKYTTNSS+DHR++LDLGLWDKFSEL+TKCIIKIVEFAKRL
Sbjct  177  LVNKVSKAHQETFPSLCQLGKYTTNSSSDHRIQLDLGLWDKFSELSTKCIIKIVEFAKRL  236

Query  240  PGFTGLTIADQITLLKAACLDILILRICTRYTPEQDTMTFSDGLTLNRTQMHNAGFGPLT  299
            PGFT LTIADQITLLK+ACLDIL+LRICTRYTPEQDTMTFSDGLTLNRTQMHNAGFGPLT
Sbjct  237  PGFTTLTIADQITLLKSACLDILMLRICTRYTPEQDTMTFSDGLTLNRTQMHNAGFGPLT  296

Query  300  DLVFTFANQLLPLEMDDTETGLLSAICLICGDRQDLEEPTKVDKLQEPLLEALKIYIRKR  359
            DLVF FA QLLPLEMDDTETGLLSAICLICGDR DLEEP +VD+LQEPLLEALKIY R+R
Sbjct  297  DLVFAFAGQLLPLEMDDTETGLLSAICLICGDRMDLEEPERVDRLQEPLLEALKIYARRR  356

Query  360  RPSKPHMFPKILMKITDLRSISAKGAERVITLKMEIPGSMPPLIQEMLENSEGHE  414
            RP+KPHMFP++LMKITDLR IS KGAER ITLKMEIPG MPPLI+EMLEN E  E
Sbjct  357  RPNKPHMFPRMLMKITDLRGISTKGAERAITLKMEIPGPMPPLIREMLENPEAFE  411
```

Supp. Figure S5: Zebrafish *rarga* is the closest homolog to the human *RARB* gene. (a) Phylogenetic analysis of human and zebrafish RA receptor genes revealed zebrafish *rarga* (DANGA) as the closest homolog to the human *RARB* gene. *RARA*, *RARB* and *RARG* represent retinoic acid receptors, alpha, beta and gamma respectively. *DRAA* and *DRAB* represent Danio rerio retinoic acid receptor's, alpha-A and alpha-B respectively. *DANGA* and *DANGB* represent Danio rerio retinoic acid receptor's, gamma-A and gamma-B respectively. (b) Identity between human RARB and zebrafish rarga protein sequences.

Supp. Table_S1. (a) List of genes included in custom capture (CC-1 and/or CC-2) high-throughput sequencing

1	*ABCA5*	34	***CRYAA***	67	*GDF5*	100	*MOBKL2C*	133	*RALDH2*	166	*TBX22*
2	***ABCB6***	35	*CTNND2*	68	***GDF6***	101	*MOBKL3*	134	*RALDH3*	167	*TEAD1*
3	***ACTB***	36	*CYB5A*	69	*GDF7*	102	*MRPL13*	135	*RARA*	168	*TEAD2*
4	***ACTG1***	37	*CYP1B1*	70	*GREM1*	103	*MST1*	136	***RARB***	169	*TEAD3*
5	*ADAMTSL4*	38	*DACT1*	71	*GREM2*	104	*MST2*	137	***RAX/RX***	170	*TEAD4*
6	*ALDH1A1*	39	*DAND5*	72	*HAXA9*	105	*NCAM1*	138	*RBP/SUGP1*	171	***TFAP2A***
7	***ALDH1A3***	40	*DCHS1*	73	*HESX1*	106	*NET1*	139	*RBP4*	172	*TMEM138*
8	*ALDH6A1*	41	*DCHS2*	74	***HMX1***	107	*NF2*	140	*RENS1*	173	*TMEM216*
9	*ALDH7A1*	42	*DHRS3*	75	***IGBP1***	108	*NKX5-3*	141	*RORB*	174	***TMEM67***
10	*ALG3*	43	*DKK1*	76	*INPP5E*	109	*NOL4*	142	*RPGRIP1L*	175	*TMX3*
11	*ANAPC1*	44	*DKK3*	77	*ITGA5*	110	*NPHP4*	143	*RPL38*	176	*TPTE*
12	*ANK2*	45	*DLL1*	78	*JAG1*	111	*NPHP9*	144	*RYBP*	177	*TWSG1*
13	*ATOH7*	46	*DPYP*	79	*KIAA0196*	112	*NR2F1*	145	*SALL1*	178	*VAV3*
14	*ATP5G1*	47	*E2F4*	80	*KLF12*	113	*NR2F2*	146	***SALL2***	179	*VAX1*
15	***BCOR***	48	*EPHA5*	81	*LAMA1*	114	*NR3C1*	147	*SALL4*	180	*VAX2*
16	*BMP2*	49	*EYA1*	82	*LAMB1*	115	***OTX2***	148	*SCRIB*	181	*WNT3*
17	*BMP3*	50	*FADD*	83	*LAMC1*	116	*PARP3*	149	***SEMA3E***	182	*WNT5A*
18	***BMP4***	51	*FAM187B*	84	*LATS1*	117	***PAX2***	150	*SFRP1*	183	WNT5B
19	***BMP7***	52	*FAT1*	85	*LATS2*	118	***PAX6***	151	*SFRP2*	184	WNT8A
20	*BMPR1B*	53	*FAT4*	86	*LHX2*	119	*PCGF5*	152	*SFRP5*	185	WNT8B
21	***C12orf57***	54	*FBXL10*	87	*LHX5*	120	***PDE6D***	153	***SHH***	186	*WW45*
22	*C5orf20*	55	*FDM6*	88	*LMX1B*	121	*PDGFA*	154	***SIX3***	187	*WWC1*
23	***CC2D2A***	56	*FGF3*	89	*LRP6*	122	*PDGFD*	155	*SKI*	188	***YAP1***
24	*CDH2*	57	*FGF8*	90	***MAB21IL2***	123	*PHACTR4*	156	*SLC16A4*	189	*ZFP503 (NLZ2)*
25	*CDH4*	58	*FLG2*	91	***MAF***	124	*PHB1*	157	*SLC38A8*	190	*ZFP703 (NLZ1)*
26	*CEP290*	59	*FOXD1*	92	*MATH5*	125	*PHB2*	158	***SMOC1***	191	*ZIC1*
27	*CER1*	60	***FOXE3***	93	*MEIS1*	126	*PIGL*	159	*SOX11*	192	*ZIC2*
28	***CHD7***	61	*FOXG1*	94	*MEIS2*	127	*PKP2*	160	***SOX2***	193	*ZIC4*
29	*CHORDIN*	62	*FREM1*	95	***MITF***	128	*PORCN*	161	*SOX6*		
30	*CHRDL1*	63	*FZ5*	96	*MKS3*	129	*POU4F2*	162	*SPECC1L*		
31	***CHX10***	64	*FZ8*	97	*MOBKL1A*	130	***PTCH1***	163	***SRD5A3***		
32	*CLDN19*	65	*GDF11*	98	*MOBKL1B*	131	*PUF60*	164	***STRA6***		
33	*CPN2*	66	***GDF3***	99	*MOBKL2a*	132	*RALDH1*	165	*TAZ*		

Genes in bold are human coloboma-associated genes.

(b) Exon-wise coverage for each coloboma-associated gene included in the sequencing panel

Gene	Chromosome	Start	End	Base Pairs	Average	Min	Max
ABCB6	chr2	220074659	220074770	111	100	100	100
ABCB6	chr2	220074949	220075022	73	100	100	100
ABCB6	chr2	220075100	220075199	99	100	100	100
ABCB6	chr2	220075430	220075547	117	100	100	100
ABCB6	chr2	220075653	220075832	179	100	100	100
ABCB6	chr2	220077106	220077215	109	100	100	100
ABCB6	chr2	220077727	220077789	62	100	100	100
ABCB6	chr2	220077960	220078050	90	100	100	100
ABCB6	chr2	220078158	220078226	68	100	100	100
ABCB6	chr2	220078309	220078390	81	100	100	100
ABCB6	chr2	220078545	220078675	130	100	100	100
ABCB6	chr2	220078840	220078910	70	100	100	100
ABCB6	chr2	220079108	220079222	114	100	100	100
ABCB6	chr2	220079680	220079806	126	100	100	100
ABCB6	chr2	220080716	220080904	188	100	99	100
ABCB6	chr2	220081083	220081189	106	100	100	100
ABCB6	chr2	220081371	220081556	185	100	100	100
ABCB6	chr2	220082389	220082531	142	100	100	100
ABCB6	chr2	220082844	220083395	551	97	48	100
ACTB	chr7	5567378	5567524	146	100	100	100
ACTB	chr7	5567632	5567818	186	100	100	100
ACTB	chr7	5567909	5568352	443	100	100	100
ACTB	chr7	5568789	5569033	244	100	100	100
ACTB	chr7	5569163	5569288	125	81	0	100
ACTB	chr7	5569294	5569296	2	81	0	100
ACTB	chr7	5570152	5570154	2	21	0	100
ACTG1	chr17	79477715	79477861	146	100	100	100
ACTG1	chr17	79477950	79478136	186	100	100	100
ACTG1	chr17	79478211	79478654	443	100	100	100
ACTG1	chr17	79478926	79479170	244	100	100	100
ACTG1	chr17	79479255	79479380	125	100	99	100
ACTG1	chr17	79479386	79479388	2	100	100	100
ACTG1	chr17	79479638	79479640	2	52	0	100
ACTG1	chr17	79479757	79479759	2	31	0	100
ALDH1A3	chr15	101420112	101420213	101	40	0	100
ALDH1A3	chr15	101425469	101425578	109	100	100	100
ALDH1A3	chr15	101427774	101427919	145	100	99	100

ALDH1A3	chr15	101432712	101432846	134	100	100	100
ALDH1A3	chr15	101433147	101433213	66	100	100	100
ALDH1A3	chr15	101434156	101434289	133	100	100	100
ALDH1A3	chr15	101436135	101436253	118	100	100	100
ALDH1A3	chr15	101438285	101438392	107	100	100	100
ALDH1A3	chr15	101440777	101440966	189	100	100	100
ALDH1A3	chr15	101445725	101445894	169	100	100	100
ALDH1A3	chr15	101447323	101447485	162	100	100	100
ALDH1A3	chr15	101448610	101448689	79	100	100	100
ALDH1A3	chr15	101454903	101454978	75	100	100	100
BCOR	chrX	39911361	39911655	294	100	100	100
BCOR	chrX	39913136	39913297	161	100	100	100
BCOR	chrX	39913506	39913588	82	100	100	100
BCOR	chrX	39914618	39914768	150	100	100	100
BCOR	chrX	39916405	39916576	171	100	100	100
BCOR	chrX	39921389	39921648	259	100	100	100
BCOR	chrX	39921996	39922326	330	100	100	100
BCOR	chrX	39922858	39923207	349	100	100	100
BCOR	chrX	39923586	39923854	268	100	100	100
BCOR	chrX	39930223	39930414	191	100	100	100
BCOR	chrX	39930887	39930945	58	100	100	100
BCOR	chrX	39931599	39934435	2836	100	100	100
BCOR	chrX	39935704	39935787	83	100	100	100
BCOR	chrX	39937094	39937182	88	100	100	100
BCOR	chrX	39937222	39937224	2	100	100	100
BCOR	chrX	39956465	39956467	2	100	50	100
BCOR	chrX	40036257	40036259	2	100	100	100
BMP7	chr20	55746014	55746166	152	100	100	100
BMP7	chr20	55748253	55748368	115	100	100	100
BMP7	chr20	55749984	55750065	81	100	100	100
BMP7	chr20	55758775	55758977	202	100	100	100
BMP7	chr20	55777528	55777681	153	100	100	100
BMP7	chr20	55803282	55803479	197	100	100	100
BMP7	chr20	55840758	55841178	420	91	49	100
C12orf57	chr12	7052812	7052827	15	99	0	100
C12orf57	chr12	7053267	7053269	2	100	100	100
C12orf57	chr12	7053284	7053338	54	100	100	100
C12orf57	chr12	7053540	7053542	2	100	100	100
C12orf57	chr12	7053636	7053817	181	100	100	100
C12orf57	chr12	7054931	7055085	154	100	100	100
CC2D2A	chr4	15471681	15471683	2	100	100	100

CC2D2A	chr4	15474843	15474845	2	100	100	100
CC2D2A	chr4	15474879	15474881	2	100	100	100
CC2D2A	chr4	15477536	15477538	2	100	100	100
CC2D2A	chr4	15477556	15477597	41	100	100	100
CC2D2A	chr4	15480344	15480432	88	100	100	100
CC2D2A	chr4	15480844	15480954	110	100	100	100
CC2D2A	chr4	15482325	15482453	128	100	100	100
CC2D2A	chr4	15482818	15482909	91	97	0	100
CC2D2A	chr4	15504049	15504142	93	100	99	100
CC2D2A	chr4	15504442	15504548	106	100	100	100
CC2D2A	chr4	15511759	15511865	106	100	100	100
CC2D2A	chr4	15512867	15513048	181	100	100	100
CC2D2A	chr4	15516327	15516494	167	100	100	100
CC2D2A	chr4	15517488	15517629	141	100	100	100
CC2D2A	chr4	15518245	15518381	136	100	100	100
CC2D2A	chr4	15529067	15529281	214	100	100	100
CC2D2A	chr4	15530240	15530351	111	100	100	100
CC2D2A	chr4	15534813	15534958	145	100	97	100
CC2D2A	chr4	15538540	15538701	161	100	100	100
CC2D2A	chr4	15539519	15539762	243	100	100	100
CC2D2A	chr4	15542457	15542639	182	100	100	100
CC2D2A	chr4	15552444	15552605	161	100	100	100
CC2D2A	chr4	15554778	15554930	152	100	99	100
CC2D2A	chr4	15556692	15556835	143	100	100	100
CC2D2A	chr4	15558924	15559132	208	100	100	100
CC2D2A	chr4	15560785	15560882	97	100	100	100
CC2D2A	chr4	15562151	15562247	96	100	100	100
CC2D2A	chr4	15564975	15565147	172	100	100	100
CC2D2A	chr4	15568997	15569107	110	100	100	100
CC2D2A	chr4	15569297	15569411	114	100	100	100
CC2D2A	chr4	15570913	15571014	101	100	100	100
CC2D2A	chr4	15572018	15572121	103	100	100	100
CC2D2A	chr4	15575770	15575951	181	100	100	100
CC2D2A	chr4	15581588	15581796	208	100	100	100
CC2D2A	chr4	15587777	15587871	94	100	100	100
CC2D2A	chr4	15589436	15589554	118	100	100	100
CC2D2A	chr4	15591165	15591304	139	100	98	100
CC2D2A	chr4	15597705	15597832	127	100	99	100
CC2D2A	chr4	15599027	15599090	63	100	100	100
CC2D2A	chr4	15601149	15601331	182	100	100	100
CC2D2A	chr4	15602857	15603048	191	100	100	100

CHD7	chr8	61591641	61591643	2	81	0	100
CHD7	chr8	61653815	61653817	2	100	100	100
CHD7	chr8	61653991	61655658	1667	100	100	100
CHD7	chr8	61693556	61693991	435	100	100	100
CHD7	chr8	61707542	61707688	146	100	100	100
CHD7	chr8	61712944	61713086	142	100	100	100
CHD7	chr8	61714084	61714154	70	100	100	100
CHD7	chr8	61720773	61720833	60	100	100	100
CHD7	chr8	61728943	61729062	119	100	99	100
CHD7	chr8	61732563	61732651	88	100	100	100
CHD7	chr8	61734346	61734488	142	100	100	100
CHD7	chr8	61734580	61734706	126	100	100	100
CHD7	chr8	61735059	61735307	248	100	99	100
CHD7	chr8	61736396	61736577	181	100	90	100
CHD7	chr8	61741219	61741367	148	100	100	100
CHD7	chr8	61742878	61743138	260	100	100	100
CHD7	chr8	61748629	61748844	215	100	100	100
CHD7	chr8	61749373	61749573	200	100	100	100
CHD7	chr8	61750224	61750396	172	100	100	100
CHD7	chr8	61750632	61750816	184	100	100	100
CHD7	chr8	61754200	61754315	115	100	100	100
CHD7	chr8	61754403	61754613	210	100	100	100
CHD7	chr8	61757420	61757624	204	100	100	100
CHD7	chr8	61757806	61757970	164	100	99	100
CHD7	chr8	61761071	61761165	94	100	100	100
CHD7	chr8	61761607	61761715	108	100	100	100
CHD7	chr8	61763049	61763183	134	100	100	100
CHD7	chr8	61763588	61763665	77	100	100	100
CHD7	chr8	61763818	61763880	62	100	100	100
CHD7	chr8	61764575	61764808	233	100	100	100
CHD7	chr8	61765054	61765267	213	100	100	100
CHD7	chr8	61765385	61766061	676	100	100	100
CHD7	chr8	61766919	61767084	165	100	100	100
CHD7	chr8	61768531	61768763	232	100	100	100
CHD7	chr8	61769001	61769449	448	100	100	100
CHD7	chr8	61773460	61773686	226	100	100	100
CHD7	chr8	61774752	61774897	145	100	100	100
CHD7	chr8	61775104	61775213	109	100	100	100
CHD7	chr8	61777572	61778492	920	100	100	100
CRYAA	chr21	44589209	44589400	191	100	100	100
CRYAA	chr21	44590624	44590751	127	100	100	100

CRYAA	chr21	44592178	44592390	212	100	99	100
FOXE3	chr1	47881985	47882949	964	43	0	100
GDF3	chr12	7842473	7843302	829	100	100	100
GDF3	chr12	7848054	7848324	270	100	100	100
GDF6	chr8	97156790	97157754	964	76	49	100
GDF6	chr8	97172512	97172920	408	97	64	100
HMX1	chr4	8847871	8848052	181	0	0	0
HMX1	chr4	8869418	8870073	655	65	5	100
HMX1	chr4	8872944	8873340	396	39	0	100
IGBP1	chrX	69353390	69353392	2	100	100	100
IGBP1	chrX	69353570	69353572	2	100	100	100
IGBP1	chrX	69353797	69353987	190	100	100	100
IGBP1	chrX	69354370	69354668	298	100	100	100
IGBP1	chrX	69366480	69366680	200	100	100	100
IGBP1	chrX	69368608	69368692	84	100	100	100
IGBP1	chrX	69370057	69370174	117	100	100	100
IGBP1	chrX	69385680	69385831	151	100	100	100
MAB21L2	chr4	151504179	151505263	1084	100	100	100
MAF	chr16	79628356	79628452	96	100	100	100
MAF	chr16	79632675	79633801	1126	75	50	100
MITF	chr3	69788748	69788854	106	100	100	100
MITF	chr3	69812843	69812845	2	100	100	100
MITF	chr3	69812992	69813095	103	100	100	100
MITF	chr3	69915441	69915499	58	100	100	100
MITF	chr3	69928282	69928536	254	100	100	100
MITF	chr3	69985873	69985908	35	100	100	100
MITF	chr3	69986970	69987202	232	100	100	100
MITF	chr3	69987500	69987517	17	100	100	100
MITF	chr3	69988246	69988334	88	100	100	100
MITF	chr3	69990384	69990484	100	100	100	100
MITF	chr3	69998199	69998321	122	100	100	100
MITF	chr3	70000960	70001039	79	100	99	100
MITF	chr3	70005603	70005683	80	100	100	100
MITF	chr3	70008421	70008573	152	100	100	100
MITF	chr3	70013995	70014399	404	100	100	100
OTX2	chr14	57268452	57269075	623	100	100	100
OTX2	chr14	57270903	57271083	180	100	100	100
OTX2	chr14	57272075	57272174	99	100	100	100
OTX2	chr14	57272293	57272295	2	100	100	100
OTX2	chr14	57276874	57276876	2	100	100	100
OTX2	chr14	57276940	57276942	2	100	100	100

OTX2	chr14	57277091	57277093	2	100	100	100
PAX2	chr10	102495465	102495492	27	0	0	0
PAX2	chr10	102505947	102506062	115	96	3	100
PAX2	chr10	102509500	102509673	173	100	47	100
PAX2	chr10	102510448	102510650	202	100	100	100
PAX2	chr10	102539252	102539342	90	100	100	100
PAX2	chr10	102541000	102541124	124	100	100	100
PAX2	chr10	102546697	102546770	73	100	100	100
PAX2	chr10	102566184	102566364	180	100	100	100
PAX2	chr10	102568864	102568995	131	100	99	100
PAX2	chr10	102584402	102584508	106	100	100	100
PAX2	chr10	102584656	102584743	87	100	100	100
PAX2	chr10	102586763	102586854	91	93	0	100
PAX2	chr10	102587297	102587440	143	100	99	100
PAX6	chr11	31811481	31811569	88	100	99	100
PAX6	chr11	31812255	31812410	155	100	100	100
PAX6	chr11	31814811	31815103	292	100	100	100
PAX6	chr11	31815197	31815352	155	100	100	100
PAX6	chr11	31815577	31815664	87	100	100	100
PAX6	chr11	31816175	31816338	163	100	100	100
PAX6	chr11	31822236	31822406	170	100	100	100
PAX6	chr11	31822775	31822777	2	99	0	100
PAX6	chr11	31823106	31823326	220	100	100	100
PAX6	chr11	31823416	31823462	46	100	100	100
PAX6	chr11	31824249	31824384	135	100	100	100
PAX6	chr11	31825497	31825499	2	0	0	0
PAX6	chr11	31827947	31827959	12	100	100	100
PAX6	chr11	31828010	31828012	2	100	100	100
PAX6	chr11	31828394	31828396	2	100	100	100
PAX6	chr11	31828473	31828475	2	100	100	100
PAX6	chr11	31832373	31832375	2	100	100	100
PAX6	chr11	31832563	31832565	2	100	100	100
PAX6	chr11	31832660	31832662	2	100	100	100
PAX6	chr11	31832758	31832760	2	100	100	100
PAX6	chr11	31833638	31833640	2	100	100	100
PAX6	chr11	31833694	31833696	2	100	100	100
PAX6	chr11	31839354	31839356	2	98	0	100
PDE6D	chr2	232597661	232597745	84	100	100	100
PDE6D	chr2	232601894	232602004	110	100	100	100
PDE6D	chr2	232602720	232602850	130	100	100	100
PDE6D	chr2	232603807	232603900	93	100	100	100

PDE6D	chr2	232645772	232645824	52	81	0	100
PTCH1	chr9	98208673	98208675	2	100	100	100
PTCH1	chr9	98209190	98209192	2	100	100	100
PTCH1	chr9	98209193	98209735	542	100	93	100
PTCH1	chr9	98211348	98211607	259	100	100	100
PTCH1	chr9	98212120	98212224	104	100	100	100
PTCH1	chr9	98215757	98215904	147	100	100	100
PTCH1	chr9	98218555	98218697	142	100	100	100
PTCH1	chr9	98220292	98220577	285	100	100	100
PTCH1	chr9	98221879	98222067	188	100	100	100
PTCH1	chr9	98224135	98224282	147	100	100	100
PTCH1	chr9	98229395	98229709	314	100	100	100
PTCH1	chr9	98231030	98231437	407	100	100	100
PTCH1	chr9	98232092	98232215	123	100	100	100
PTCH1	chr9	98238313	98238443	130	100	99	100
PTCH1	chr9	98239038	98239141	103	100	100	100
PTCH1	chr9	98239826	98239986	160	100	100	100
PTCH1	chr9	98240334	98240470	136	100	100	100
PTCH1	chr9	98241279	98241431	152	100	100	100
PTCH1	chr9	98242248	98242374	126	100	100	100
PTCH1	chr9	98242669	98242872	203	100	100	100
PTCH1	chr9	98244228	98244324	96	100	100	100
PTCH1	chr9	98244413	98244487	74	100	100	100
PTCH1	chr9	98247964	98248158	194	100	100	100
PTCH1	chr9	98268686	98268883	197	100	100	100
PTCH1	chr9	98269216	98269218	2	100	100	100
PTCH1	chr9	98269508	98269510	2	100	100	100
PTCH1	chr9	98270213	98270215	2	100	100	100
PTCH1	chr9	98270230	98270232	2	100	100	100
PTCH1	chr9	98270440	98270643	203	70	41	100
PTCH1	chr9	98278748	98278753	5	100	100	100
PTCH1	chr9	98278902	98279102	200	100	100	100
RARB	chr3	24870894	24870896	2	0	0	0
RARB	chr3	24900162	24900164	2	0	0	0
RARB	chr3	24900243	24900245	2	0	0	0
RARB	chr3	25101613	25101615	2	0	0	0
RARB	chr3	25101667	25101669	2	0	0	0
RARB	chr3	25215607	25215609	2	100	100	100
RARB	chr3	25215888	25216068	180	100	100	100
RARB	chr3	25382354	25382384	30	100	100	100
RARB	chr3	25470012	25470024	12	100	100	100

RARB	chr3	25470222	25470381	159	100	100	100
RARB	chr3	25499884	25499886	2	100	100	100
RARB	chr3	25502681	25502834	153	100	100	100
RARB	chr3	25542670	25542816	146	100	100	100
RARB	chr3	25611246	25611411	165	100	100	100
RARB	chr3	25622034	25622215	181	100	100	100
RARB	chr3	25634991	25635200	209	100	100	100
RARB	chr3	25636008	25636171	163	100	100	100
RARB	chr3	25637908	25638107	199	100	100	100
RAX	chr18	56936235	56936735	500	69	7	100
RAX	chr18	56939590	56939848	258	98	83	100
RAX	chr18	56940147	56940438	291	74	0	100
SALL2	chr14	21990141	21990380	239	100	100	100
SALL2	chr14	21990837	21993790	2953	100	100	100
SALL2	chr14	21994192	21994261	69	96	0	100
SALL2	chr14	22004980	22005055	75	100	100	100
SEMA3E	chr7	82996901	82997356	455	100	100	100
SEMA3E	chr7	83014607	83014751	144	100	100	100
SEMA3E	chr7	83016296	83016368	72	100	99	100
SEMA3E	chr7	83021868	83022039	171	100	100	100
SEMA3E	chr7	83023610	83023656	46	100	100	100
SEMA3E	chr7	83025951	83026047	96	100	99	100
SEMA3E	chr7	83029341	83029568	227	100	100	100
SEMA3E	chr7	83031945	83032094	149	100	99	100
SEMA3E	chr7	83034763	83034837	74	100	99	100
SEMA3E	chr7	83035258	83035377	119	100	99	100
SEMA3E	chr7	83036410	83036557	147	100	99	100
SEMA3E	chr7	83037681	83037805	124	100	98	100
SEMA3E	chr7	83047703	83047801	98	100	100	100
SEMA3E	chr7	83095795	83095919	124	100	100	100
SEMA3E	chr7	83098556	83098620	64	100	100	100
SEMA3E	chr7	83119427	83119592	165	100	72	100
SEMA3E	chr7	83270645	83270647	2	100	100	100
SEMA3E	chr7	83277741	83277858	117	100	98	100
SHH	chr7	155592700	155592941	241	0	0	0
SHH	chr7	155595593	155596422	829	63	0	100
SHH	chr7	155598987	155599253	266	100	100	100
SHH	chr7	155599402	155599443	41	97	0	100
SHH	chr7	155599533	155599535	2	93	0	100
SHH	chr7	155601684	155601686	2	0	0	0
SHH	chr7	155604514	155604816	302	100	100	100

SIX3	chr2	45169243	45170051	808	93	51	100
SIX3	chr2	45171704	45171899	195	88	54	100
SMOC1	chr14	70346395	70346496	101	91	0	100
SMOC1	chr14	70418852	70419022	170	100	100	100
SMOC1	chr14	70420134	70420251	117	100	100	100
SMOC1	chr14	70442429	70442533	104	100	100	100
SMOC1	chr14	70444632	70444684	52	100	100	100
SMOC1	chr14	70459131	70459192	61	100	100	100
SMOC1	chr14	70461114	70461199	85	100	100	100
SMOC1	chr14	70477468	70477665	197	100	100	100
SMOC1	chr14	70478199	70478286	87	100	100	100
SMOC1	chr14	70480100	70480210	110	100	100	100
SMOC1	chr14	70489917	70490166	249	100	100	100
SMOC1	chr14	70496956	70496975	19	100	100	100
SOX2	chr3	181430146	181431104	958	97	72	100
SRD5A3	chr4	56212503	56212726	223	80	0	100
SRD5A3	chr4	56225510	56225657	147	100	100	100
SRD5A3	chr4	56230238	56230440	202	100	100	100
SRD5A3	chr4	56233752	56233891	139	100	100	100
SRD5A3	chr4	56235996	56236258	262	100	100	100
STRA6	chr15	74472420	74472586	166	100	100	100
STRA6	chr15	74473120	74473280	160	100	100	100
STRA6	chr15	74473633	74473801	168	100	100	100
STRA6	chr15	74474499	74474605	106	100	100	100
STRA6	chr15	74474681	74474803	122	100	100	100
STRA6	chr15	74476194	74476332	138	100	100	100
STRA6	chr15	74477318	74477398	80	100	100	100
STRA6	chr15	74481453	74481620	167	100	100	100
STRA6	chr15	74483178	74483244	66	100	100	100
STRA6	chr15	74483505	74483586	81	100	100	100
STRA6	chr15	74483762	74483834	72	100	100	100
STRA6	chr15	74486138	74486265	127	100	100	100
STRA6	chr15	74487640	74487811	171	100	100	100
STRA6	chr15	74487942	74488018	76	100	100	100
STRA6	chr15	74488346	74488490	144	100	100	100
STRA6	chr15	74489676	74489766	90	100	99	100
STRA6	chr15	74490090	74490161	71	100	100	100
STRA6	chr15	74494493	74494625	132	100	100	100
STRA6	chr15	74494756	74494860	104	100	100	100
STRA6	chr15	74495051	74495053	2	100	100	100
STRA6	chr15	74495341	74495343	2	100	100	100

STRA6	chr15	74500035	74500067	32	100	100	100
STRA6	chr15	74501138	74501140	2	100	100	100
STRA6	chr15	74501697	74501795	98	100	100	100
TFAP2A	chr6	10398649	10398940	291	100	100	100
TFAP2A	chr6	10400678	10400824	146	100	100	100
TFAP2A	chr6	10402722	10402845	123	100	100	100
TFAP2A	chr6	10404738	10404974	236	100	100	100
TFAP2A	chr6	10407023	10407079	56	100	100	100
TFAP2A	chr6	10410131	10410570	439	100	88	100
TFAP2A	chr6	10411800	10411829	29	100	100	100
TFAP2A	chr6	10415171	10415218	47	100	100	100
TFAP2A	chr6	10419648	10419683	35	100	100	100
TMEM67	chr8	94767137	94767139	2	100	100	100
TMEM67	chr8	94767142	94767367	225	100	100	100
TMEM67	chr8	94768003	94768096	93	100	98	100
TMEM67	chr8	94770708	94770806	98	100	100	100
TMEM67	chr8	94772085	94772223	138	100	95	100
TMEM67	chr8	94776067	94776171	104	100	100	100
TMEM67	chr8	94777631	94777705	74	100	99	100
TMEM67	chr8	94777797	94777876	79	100	100	100
TMEM67	chr8	94784814	94784881	67	100	99	100
TMEM67	chr8	94792818	94792977	159	100	100	100
TMEM67	chr8	94793099	94793212	113	100	73	100
TMEM67	chr8	94793883	94793974	91	100	100	100
TMEM67	chr8	94794620	94794690	70	100	100	100
TMEM67	chr8	94797447	94797608	161	100	100	100
TMEM67	chr8	94798448	94798576	128	100	100	100
TMEM67	chr8	94800069	94800179	110	100	100	100
TMEM67	chr8	94803488	94803549	61	100	64	100
TMEM67	chr8	94805423	94805526	103	100	100	100
TMEM67	chr8	94807634	94807737	103	100	100	100
TMEM67	chr8	94808126	94808217	91	100	99	100
TMEM67	chr8	94809359	94809463	104	100	100	100
TMEM67	chr8	94809556	94809700	144	100	100	100
TMEM67	chr8	94811843	94811988	145	100	100	100
TMEM67	chr8	94815829	94815914	85	100	100	100
TMEM67	chr8	94816987	94817108	121	100	100	100
TMEM67	chr8	94821065	94821186	121	100	100	100
TMEM67	chr8	94821282	94821391	109	100	72	100
TMEM67	chr8	94822010	94822117	107	100	100	100
TMEM67	chr8	94827530	94827677	147	100	100	100

TMEM67	chr8	94828597	94828680	83	100	100	100
VSX2	chr14	74706264	74706636	372	91	38	100
VSX2	chr14	74707882	74707971	89	100	100	100
VSX2	chr14	74711865	74711993	128	100	100	100
VSX2	chr14	74726302	74726487	185	100	100	100
VSX2	chr14	74727294	74727622	328	100	100	100
YAP1	chr11	101981579	101981902	323	71	0	100
YAP1	chr11	101983476	101983478	2	100	100	100
YAP1	chr11	101984872	101985127	255	100	100	100
YAP1	chr11	102033184	102033304	120	100	100	100
YAP1	chr11	102056746	102056864	118	100	99	100
YAP1	chr11	102076621	102076819	198	100	100	100
YAP1	chr11	102080245	102080297	52	100	100	100
YAP1	chr11	102094350	102094485	135	100	100	100
YAP1	chr11	102098197	102098314	117	100	100	100
YAP1	chr11	102100430	102100671	241	100	100	100

Supp. Table S1: (a) List of genes included in custom capture (CC-1 and/or CC-2) high-throughput sequencing. Genes in bold are human coloboma-associated genes. **(b) Exon-wise coverage for each coloboma-associated gene included in the sequencing panel.** The “Start” and “End” numbers represent the coordinates for each exon in the gene. The data is showed as average, minimum and maximum coverage generated from the sequencing data for all the subjects included in the panel.

Supp. Table_S2: RARB Protein Conservation Across Vertebrate Species (PROMALS3D Alignment):

RARB Domain regions in Human RARB protein:

http://pfam.xfam.org/protein/P10826

		Start - End
1)	Low Complexity -	33 - 51
2)	Low Complexity –	66 - 81
3)	Zf-C4 (DBD) – (88 - 108, 124 - 143)	86 - 155
4)	Coiled coil –	177 - 197
5)	Hormone Receptor(LBD) - (183-417 - NR-LBD)	224 - 401
6)	Low Complexity –	434 - 451

Conservation:
NP 001277145.1 retinoic a: Human
XP_028699464.1_retinoic_a: Rhesus
NP_001276689.1_retinoic_a: Mouse
XP_003433175.1_retinoic_a: Dog
XP_003415774.1_retinoic_a: Elephant
NP_990657.1_retinoic_acid: Chicken
XP_002932450.2_PREDICTED: Xenopus
NP_571414.1_retinoic_acid: Zebrafish
tr_S4RRL8_S4RRL8_PETMA_Re: Lamprey

```
Conservation:                    95555   9555555555555555555555556  9   6 6 6  655555556 66  55555555555
NP_001277145.1_retinoic_a    1   MTTSGHACPVPAVNGHMTHYPATPYPLLFPPVIGGLSLPPLHGLHGHPPPSGCSTPSPA-----------   59
XP_028699464.1_retinoic_a    1   MTTSGHACPVPAVNGHMTHYPATPYPLLFPPVIGGLSLPPLHGLHGHPPPSGCSTPSPA-----------   59
NP_001276689.1_retinoic_a    1   MSTSSHACPVPAVRGHMTHYPAAPYPLLFPPVIRGLSLPPLHGLHGHPPPSGCSTPSPASVGQACQRTTG   70
XP_003433175.1_retinoic_a    1   MTTSSHACPVPAVNGHMTHYPAAPYPLLFPPVIGGLSLPPLHGLHGHPPPSGCSTPSPA-----------   59
XP_003415774.1_retinoic_a    1   M----FDC------------------------MDVLSLSPGQILDFY------- TASPSSCMLQEKALKA   35
NP_990657.1_retinoic_acid    1   MTTSSRTCPVPAVNGHMTHYPAAPYPLLFPPVIGGLSLPSLHGLQSHPPTSGCSTPSPA-----------   59
XP_002932450.2_PREDICTED:    1   M----FDC------------------------MDVLAVSPGQMLDFY------- TASPSSCMLQEKALKA   35
NP_571414.1_retinoic_acid    1   M----FDC------------------------MEALGMGPRQLYD--------- VTNRGACMLRKAS--P   31
tr_S4RRL8_S4RRL8_PETMA_Re        ----------------------------------------------------------------------
Consensus_aa:                    M....@sC........................h.sLthsP.p.hc.@.......hss.t...........
Consensus_ss:
```

```
Conservation:                    55555555555555555   6999999996669969996999969999999999999999999699999999
NP_001277145.1_retinoic_a   60   -----------------TIETQSTSSEELVPSPPSPLPPPRVYKPCFVCQDKSSGYHYGVSACEGCKGFF   112
XP_028699464.1_retinoic_a   60   -----------------TIETQSTSSEELVPSPPSPLPPPRVYKPCFVCQDKSSGYHYGVSACEGCKGFF   112
NP_001276689.1_retinoic_a   71   GSQFAASTKWTPSLNA-AIETQSTSSEELVPSPPSPLPPPRVYKPCFVCQDKSSGYHYGVSACEGCKGFF   139
XP_003433175.1_retinoic_a   60   -----------------TIETQSTSSEELVPSPPSPLPPPRVYKPCFVCQDKSSGYHYGVSACEGCKGFF   112
XP_003415774.1_retinoic_a   36   CFSGITQTEWQHQHTAQSIETQSTSSEELLPSPPSPLPPPRVYKPCFVCQDKSSGYHYGVSACEGCKGFF   105
NP_990657.1_retinoic_acid   60   -----------------TVETQSTSSEELVPSPPSPLPPPRVYKPCFVCQDKSSGYHYGVSACEGCKGFF   112
XP_002932450.2_PREDICTED:   36   CFNGLAQTDWQHRHSAQSIETQSTSSEDLVPSPPSPPPPPRIYKPCFVCQDKSSGYHYGVSACEGCKGFF   105
NP_571414.1_retinoic_acid   32   FYAGLDPFAWTGTASVRSVETQSTSSEEMVPSSPSPPPPPRVYKPCFVCQDKSSGYHYGVSSCEGCKGFF   101
tr_S4RRL8_S4RRL8_PETMA_Re        ----------------------------------------------------------------------
Consensus_aa:                    .................olETQSTSSEEhVPSsPSP.PPPRVYKPCFVCQDKSSGYHYGVStCEGCKGFF
Consensus_ss:                                        hhh                   ee             hhhh
```

```
Conservation:                     99999999699999699969999999999999999699999999969999999999996 6 6 5 969966
NP_001277145.1_retinoic_a   113   RRSIQKNMIYTCHRDKNCVINKVTRNRCQYCRLQKCFEVGMSKESVRNDRNKKKKETSKQEC-TESYEMT    181
XP_028699464.1_retinoic_a   113   RRSIQKNMIYTCHRDKNCVINKVTRNRCQYCRLQKCFEVGMSKESVRNDRNKKKKETSKQEC-TESYEMT    181
NP_001276689.1_retinoic_a   140   RRSIQKNMIYTCHRDKNCVINKVTRNRCQYCRLQKCFEVGMSKESVRNDRNKKKKEPSKQEC-TESYEMT    208
XP_003433175.1_retinoic_a   113   RRSIQKNMIYTCHRDKNCVINKVTRNRCQYCRLQKCFEVGMSKESVRNDRNKKKKEPSKQEC-TESYEMT    181
XP_003415774.1_retinoic_a   106   RRSIQKNMIYTCHRDKNCVINKVTRNRCQYCRLQKCFEVGMSKESVRNDRNKKKKEPSKQEC-TESYEMT    174
NP_990657.1_retinoic_acid   113   RRSIQKNMVYTCHRDKNCVINKVTRNRCQYCRLQKCFEVGMSKESVRNDRNKKKKEPTKQES-TENYEMT    181
XP_002932450.2_PREDICTED:   106   RRSIQKNMVYTCHREKNCVINKVTRNRCQYCRLQRCFEVGMSKESVRNDRNKKKKEPSKIEC-IENYEMT    174
NP_571414.1_retinoic_acid   102   RRSIQKNMVYTCHRDKNCQINKVTRNRCQYCRLQKCFEVGMSKEAVRNDRNKKKKDVKDEVIPPESYELS    171
tr_S4RRL8_S4RRL8_PETMA_Re         ----------------------------------------------------------------------
Consensus aa:                     RRSIQKNMlYTCHRDKNC.INKVTRNRCQYCRLQKCFEVGMSKEtVRNDRNKKKK-spcp.h.sEsYEho
Consensus ss:                     eeee     eee                         hhhhhh

Conservation:                     6996696666699999999999999999999996669669999999999996999999999999999996
NP_001277145.1_retinoic_a   182   AELDDLTEKIRKAHQETFPSLCQLGKYTTNSSADHRVRLDLGLWDKFSELATKCIIKIVEFAKRLPGFTG    251
XP_028699464.1_retinoic_a   182   AELDDLTEKIRKAHQETFPSLCQLGKYTTNSSADHRVRLDLGLWDKFSELATKCIIKIVEFAKRLPGFTG    251
NP_001276689.1_retinoic_a   209   AELDDLTEKIRKAHQETFPSLCQLGKYTTNSSADHRVRLDLGLWDKFSELATKCIIKIVEFAKRLPGFTG    278
XP_003433175.1_retinoic_a   182   AELDDLTEKIRKAHQETFPSLCQLGKYTTNSSADHRVRLDLGLWDKFSELATKCIIKIVEFAKRLPGFTG    251
XP_003415774.1_retinoic_a   175   AELDDLTETIRKAHQETFPSLCQLGKYTTNSSADHRVRLDLGLWDKFSELATKCIIKIVEFAKRLPGFTG    244
NP_990657.1_retinoic_acid   182   AELDDLTEKIRKAHQETFPSLCQLGKYTTNSSADHRVRLDLGLWDKFSELATKCIIKIVEFAKRLPGFTS    251
XP_002932450.2_PREDICTED:   175   AELDDLTEKIRKAHQETFPSLCQLGKYTTNSSAEQRVRLDLGLWDKFSELATKCIIKIVEFAKRLPGFTS    244
NP_571414.1_retinoic_acid   172   GELEELVNKVSKAHQETFPSLCQLGKYTTNSSSDHRIQLDLGLWDKFSELSTKCIIKIVEFAKRLPGFTT    241
tr_S4RRL8_S4RRL8_PETMA_Re     1   -----------------------------NSSADHRVQLDLGLWDKFSELATKCIIKIVEFAKRLPGFVN     41
Consensus aa:                     tEL--LhpKlpKAHQETFPSLCQLGKYTTNSStDHRlpLDLGLWDKFSELtTKCIIKIVEFAKRLPGFhs
Consensus ss:                                                               hhhhhhhhhhhhhhhhhhhhhhh

Conservation:                     9699999999969999996999969999999999999999999999999999999999996996999966
NP_001277145.1_retinoic_a   252   LTIADQITLLKAACLDILILRICTRYTPEQDTMTFSDGLTLNRTQMHNAGFGPLTDLVFTFANQLLPLEM    321
XP_028699464.1_retinoic_a   252   LTIADQITLLKAACLDILILRICTRYTPEQDTMTFSDGLTLNRTQMHNAGFGPLTDLVFTFANQLLPLEM    321
NP_001276689.1_retinoic_a   279   LTIADQITLLKAACLDILILRICTRYTPEQDTMTFSDGLTLNRTQMHNAGFGPLTDLVFTFANQLLPLEM    348
XP_003433175.1_retinoic_a   252   LTIADQITLLKAACLDILILRICTRYTPEQDTMTFSDGLTLNRTQMHNAGFGPLTDLVFTFANQLLPLEM    321
XP_003415774.1_retinoic_a   245   LTIADQITLLKAACLDILILRICTRYTPEQDTMTFSDGLTLNRTQMHNAGFGPLTDLVFTFANQLLPLEM    314
NP_990657.1_retinoic_acid   252   LTIADQITLLKAACLDILILRICTRYTPEQDTMTFSDGLTLNRTQMHNAGFGPLTDLVFTFANQLLPLEM    321
XP_002932450.2_PREDICTED:   245   LTIADQITLLKAACLDILILRICTRYTPEQDTMTFSDGLTLNRTQMHNAGFGPLTDLVFTFANQLLPLEM    314
NP_571414.1_retinoic_acid   242   LTIADQITLLKSACLDILMLRICTRYTPEQDTMTFSDGLTLNRTQMHNAGFGPLTDLVFAFAGQLLPLEM    311
tr_S4RRL8_S4RRL8_PETMA_Re    42   LSIADQITLLKAACLDILILRICSRYTPEQDTMTFSDGLTLNRTQMHNAGFGPLTDLVFAFANQLLPLSV    111
Consensus aa:                     LoIADQITLLKtACLDILhLRICoRYTPEQDTMTFSDGLTLNRTQMHNAGFGPLTDLVFhFAsQLLPLph
Consensus ss:                       hhhhhhhhhhhhhhhhhhhhhhhh      eeee     ee  hhhhhhhhhhhhhhhhhhhhhhhh

Conservation:                      99996999699996 9 99699999 6966999999996969 96999 9999996 99999999699699
NP_001277145.1_retinoic_a   322    DDTETGLLSAICLICGDRQDLEEPTKVDKLQEPLLEALKIYIRKRRPSKPHMFPKILMKITDLRSISAKG   391
XP_028699464.1_retinoic_a   322    DDTETGLLSAICLICGDRQDLEEPTKVDKLQEPLLEALKIYIRKRRPSKPHMFPKILMKITDLRSISAKG   391
NP_001276689.1_retinoic_a   349    DDTETGLLSAICLICGDRQDLEEPTKVDKLQEPLLEALKIYIRKRRPSKPHMFPKILMKITDLRSISAKG   418
XP_003433175.1_retinoic_a   322    DDTETGLLSAICLICGDRQDLEEPTKVDKLQEPLLEALKIYIRKRRPSKPHMFPKILMKITDLRSISAKG   391
XP_003415774.1_retinoic_a   315    DDTETGLLSAICLICGDRQDLEEPTKVDKLQEPLLEALKIYIRKRRPSKPHMFPKILMKITDLRSISAKG   384
NP_990657.1_retinoic_acid   322    DDTETGLLSAICLICGDRQDLEEPMKVDKLQEPLLEALKIYIRKRRPNKPHMFPKILMKITDLRSISAKG   391
XP_002932450.2_PREDICTED:   315    DDTETGLLSAICLICEDRQDLEEPAKVDKLQEPLLEALKIYIRKRRPNKPHMFPKILMKITDLRSISAKG   384
NP_571414.1_retinoic_acid   312    DDTETGLLSAICLICGDRMDLEEPERVDRLQEPLLEALKIYARRRRPNKPHMFPRMLMKITDLRGISTKG   381
tr_S4RRL8_S4RRL8_PETMA_Re   112    DDTEAGLLAAICLMCADRQDLEEPEKVERLQEPLLEAMKVYVRRRRPDKPHMFPKLLMKITDLRGISAKG   181
Consensus aa:                      DDTEhGLLtAICLhCtDRbDLEEP.+V-+LQEPLLEAhKlYhR+RRPsKPHMFP+hLMKITDLRtIShKG
Consensus ss:                       hhhhhhhhhhhhh           hhhhhhhhhhhhhhhhhhhhh     hhhhhhhhhhhhhhhhhhhhh

Conservation:                     699699996999969999969999966 6 9              6   66 6        55555555555
NP_001277145.1_retinoic_a   392   AERVITLKMEIPGSMPPLIQEMLENSEGHEPLTPSSSGNTAEHSPSISPSSVENSGVSQ-----------    450
XP_028699464.1_retinoic_a   392   AERVITLKMEIPGSMPPLIQEMLENSEGHEPLTPSSSGNTAEHSPSISPSSVENSGVSQ-----------    450
NP_001276689.1_retinoic_a   419   AERVITLKMEIPGSMPPLIQEMLENSEGHEPLTPSSSGNIAEHSPSVSPSSVENSGVSQ-----------    477
XP_003433175.1_retinoic_a   392   AERVITLKMEIPGSMPPLIQEMLENSEGHEPLTPSSSGNPAEHSPSISPSSVENSGVSQ-----------    450
XP_003415774.1_retinoic_a   385   AERVITLKMEIPGSMPPLIQEMLENSEGHEPLTPSSSGNTAEHSPSISSSSAENNGVSQ-----------    443
NP_990657.1_retinoic_acid   392   AERVITLKMEIPGSMPPLIQEMLENSEGHEPLTPTSNGNTAEHSPSISPSSVDNSSVSQ-----------    450
XP_002932450.2_PREDICTED:   385   AERVITLKLEIPGSMPPLIQEMLENSEGHEASASSPSEDKTEHSHCISPSS-EEECVSK-----------    442
NP_571414.1_retinoic_acid   382   AERAITLKMEIPGPMPPLIREMLENPEAFEDQSEATEKKPEPEPPAPPPPALLTMKKEQEDEDDSWATEN    451
tr_S4RRL8_S4RRL8_PETMA_Re   182   MERVITLKMEIPGSMPPLIQEMLENSYGSEAGGTTSGSSSPGTPEATPPTAGTPEPAHD-----------    240
Consensus aa:                     hERhITLKMEIPGsMPPLIpEMLENsbt.Es.s.so..ps..ps.t.sPst..s...pp..........
Consensus ss:                     hhhhhhhh        hhhhhhhh

Conservation:                     5555 6   5555555555555555555555555555555555555
NP_001277145.1_retinoic_a   451   ----SPLVQ-------------------------------------   455
XP_028699464.1_retinoic_a   451    ----SPLVQ-------------------------------------  455
NP_001276689.1_retinoic_a   478    ----SPLLQ-------------------------------------  482
```

```
XP_003433175.1_retinoic_a   451   ----SPLVQ---------------------------------------  455
XP_003415774.1_retinoic_a   444   ----SPRVQ---------------------------------------  448
NP_990657.1_retinoic_acid   451   ----SPMVQ---------------------------------------  455
XP_002932450.2_PREDICTED:   443   ----CAQVQ---------------------------------------  447
NP_571414.1_retinoic_acid   452   GSEPSPEEEDDDDEDGEEERGTDSDGEAWGGQEPNADVSRKSHGGRAQ  499
tr_S4RRL8_S4RRL8_PETMA_Re   241   ----APHGKG--------------------------------------  246
Consensus_aa:                     ....tP..p.......................................
Consensus_ss:
```

PROMAL3D Alignment parameters for RARB protein sequences of different vertebrate species:

Alignment parameters:

Identity threshold above which fast alignment is applied: **0.6**

Weight for constraints derived from sequences: **1**

Weight for constraints derived from homologs with structures: **1.5**

Weight for constraints derived from input structures: **1.5**

Parameters for profile-profile comparison:

Weight for amino acid scores: **0.8**

Weight for predicted secondary structure scores: **0.2**

Parameters for deriving sequence profiles from PSI-BLAST searches:

PSI-BLAST iteration number: **3**

PSI-BLAST e-value inclusion threshold: **0.001**

Identity cutoff below which distant homologs are removed: **0.25**

Maximum number of homologs kept for PSI-BLAST alignment: **300**

Parameters for detecting and using homologs with 3D structures (homolog3d):

PSI-BLAST e-value cutoff against structural database: **0.001**

Identity cutoff below which 3D structures are not used: **0.2**

Align homologs with 3D structures by programs: **dali - not used; fast - used; tmalign - used**

Realign target-homolog3d using profile-profile alignment: **yes**

Parameters for pairwise alignments between input 3D structures:

Align input structures by programs: **dali - not used; fast - used; tmalign - used**

Parameters for aligning sequences within groups in the first alignment stage:

Align sequences within groups in the first alignment stage by: **mafft**

Supp. Table S2: RARB protein conservation across vertebrate species (PROMALS3D Alignment). The location of specific domains in the protein are indicated as “Start” and “End”, and the different vertebrate species included are listed. The alignment parameters are listed at the end of the document.

Supp. Table_S3. *rarga* morpholino injections and human *RARB* mRNA rescue experiments

Type of Injection	No Col / Grade 1 No. of Emb (%)	Mild Col / Grade 2 No. of Emb (%)	Severe Col / Grade 3 No. of Emb (%)	Total Emb Injected
rarga MO	61 (35)	55 (31)	60 (34)	176
rarga MO + *RARB* mRNA	81 (61)	26 (20)	26 (19)	133
rarga MO + *RARB*-Mutant mRNA	65 (44)	33 (22)	49 (33)	147
RARB mRNA	174 (100)	0 (0)	0 (0)	174
RARB-Mutant mRNA	174 (100)	0 (0)	0 (0)	174
Std_C MO	199 (100)	0 (0)	0 (0)	199
Uninj Emb	288 (100)	0 (0)	0 (0)	288

MO, Morpholino; Std_C, Standard Control; Uninj, Uninjected; Emb, Embryos; Col, Coloboma

Supp. Table S3: Zebrafish embryos injected with *rarga* SB MO and rescue experiments with human wild type *RARB* and *RARB*-mutant mRNA. MO injected at 7.5 ng/µl and mRNA injected at 150 pg/nl.
